# Protective effects of beta-blockers against anthracycline- and trastuzumab-related cardiotoxicity: a systematic review based on conventional and Bayesian network meta-analysis

**DOI:** 10.3389/fcvm.2026.1777908

**Published:** 2026-04-01

**Authors:** Shenghao Jiang, Gan Gao

**Affiliations:** School of Clinical Medicine, Faculty of Medicine and Health, The University of New South Wales, Kensington, NSW, Australia

**Keywords:** Bayesian network meta-analysis, beta-blockers, cardiotoxicity, chemotherapy, systematic review

## Abstract

**Objective:**

This study aimed to systematically evaluate the efficacy of beta-blockers (BBs) in preventing left ventricular dysfunction (LVD) induced by anthracyclines and trastuzumab. It combines traditional meta-analysis with Bayesian network meta-analysis (*N*MA) to compare the cardiac protective effects of different BBs.

**Methods:**

We conducted a systematic literature search in databases such as PubMed, Web of Science, EMBASE, and the Cochrane Library to identify eligible randomized controlled trials (RCTs). Traditional meta-analysis and Bayesian NMA were used to evaluate the impact of BBs on left ventricular ejection fraction (LVEF) and cancer therapy-related cardiotoxicity events (CTRCE). The cumulative ranking probabilities (SUCRA) were used to rank the efficacy of different BBs. Data analysis was performed using STATA 16.0 and R 4.5.0 software.

**Results:**

A total of 19 RCTs were included for LVEF outcomes and 15 for CTRCE outcomes. For LVEF, BBs were superior to controls overall (WMD = 2.19, 95% CI 1.40–2.98; *I*^2^ = 93.5%). Subgroup analysis indicated consistent and greater improvement with bisoprolol and carvedilol. NMA-SUCRA ranking: bisoprolol 0.7696, carvedilol 0.6822, nebivolol 0.6023, metoprolol 0.2413, placebo 0.2047. For CTRCEs, BBs significantly reduced event risk (RR = 0.54, 95% CI 0.44–0.67; *I*^2^ = 41.4%). Subgroup analyses showed benefit with bisoprolol (RR = 0.28, 0.15–0.50), nebivolol (RR = 0.48, 0.27–0.86), and carvedilol (RR = 0.68, 0.53–0.87), while metoprolol did not reach significance (RR = 0.33, 0.07–1.56). Treatment-specific subgroups revealed significant benefit in anthracycline (RR = 0.52, 0.37–0.74) and combination regimens (RR = 0.58, 0.45–0.79), but not in trastuzumab monotherapy (RR = 0.16, 0.02–1.26). NMA-SUCRA ranking: bisoprolol 0.8085, metoprolol 0.7303, nebivolol 0.5124, carvedilol 0.3743, placebo 0.0745.

**Conclusion:**

BBs demonstrated robust protective effects in maintaining LVEF and reducing CTRCE risk, with bisoprolol showing the greatest efficacy, followed by carvedilol. The evidence supports prioritizing BBs during chemotherapy with individualized selection based on treatment regimen and patient profile. However, large-scale, multicenter RCTs with long-term follow-up are warranted to confirm long-term benefits and to explore combination strategies with other cardioprotective agents.

**Systematic Review Registration:**

identifier CRD420251233424.

## 1 Introduction

With the advancement of cancer therapies, particularly anthracyclines and trastuzumab, survival rates have significantly improved (1). However, these agents are associated with substantial cardiotoxicity (2, 3). Cancer therapy–related cardiotoxicity (CTRC), most commonly manifested as left ventricular dysfunction (LVD), can limit chemotherapy intensity and adversely affect long-term outcomes (4). It typically begins with myocardial injury and a decline in left ventricular ejection fraction (LVEF), and may progress to clinical heart failure in severe cases (5). In addition to LVEF reduction, cancer therapy–related cardiotoxicity events (CTRCEs), such as heart failure, arrhythmias, and acute myocardial injury, further compromise prognosis. Therefore, preventing cardiac injury without reducing antitumor efficacy has become a central challenge in contemporary cardio-oncology.

Beta-blockers (BBs), as classic cardioprotective agents, are widely used in the treatment of heart failure, hypertension, and ischemic heart disease due to their ability to suppress sympathetic activation, reduce heart rate, and lower myocardial oxygen consumption (6, 7). Some BBs also possess antioxidant, anti-apoptotic, and endothelial function–enhancing properties, providing a biological basis for their potential role in preventing chemotherapy-induced cardiotoxicity (8–10).

Recent years have witnessed numerous clinical studies evaluating the efficacy of BBs in preventing chemotherapy-related declines in LVEF. However, substantial differences remain across individual BBs, and a comprehensive comparison is still lacking. Some studies have reported that BBs can attenuate the decline in LVEF (11, 12). Nevertheless, heterogeneity in drug type, dosage, treatment duration, and follow-up periods has led to inconsistent conclusions. For instance, some studies have demonstrated significant cardioprotective effects of BBs in anthracycline- or trastuzumab-induced cardiotoxicity (13, 14), while another study did not observe statistically significant benefit (15). This indicates suggest that differences in drug properties and patient heterogeneity may be key contributors to the inconsistent findings.

This study systematically integrates evidence from previous randomized controlled trials (RCTs). Unlike previous meta-analyses that mostly focused on single-drug or pairwise comparisons, this study employs both traditional and Bayesian network meta-analysis (NMA) to compare the relative efficacy of different BBs. Through traditional pairwise meta-analysis, it aims to clarify the overall protective effect of BBs on left ventricular function compared to placebo or standard treatment. Additionally, Bayesian NMA is used to further compare the relative efficacy of different BBs and identify potential optimal interventions based on cumulative ranking probabilities (SUCRA). The findings of this study are expected to provide evidence-based support for preventing chemotherapy-related cardiac toxicity and the clinical application of BBs.

## 2 Methods

This study was conducted in strict accordance with the Preferred Reporting Items for Systematic Reviews and Meta-Analyses (PRISMA) guidelines (16) and has been registered in the PROSPERO international prospective systematic review database (Registration number: CRD420251233424).

### 2.1 Search strategy

A systematic literature search was performed across PubMed, Web of Science, EMBASE, and the Cochrane Library. A comprehensive search strategy was developed using Boolean operators to ensure the inclusion of all relevant studies published up to September 24, 2025. The search strategy incorporated both Medical Subject Headings (MeSH) and free-text terms, focusing primarily on concepts such as “Adrenergic beta-Antagonists,” “Trastuzumab,” “Anthracyclines,” and “Ventricular Dysfunction.” In addition, the reference lists of included studies and relevant reviews were manually screened to identify potentially eligible trials. The complete search strategy is provided in Supplementary Material S1.

### 2.2 Inclusion and exclusion criteria

The studies included in this study must meet the following criteria: (1) adult patients diagnosed with malignant tumors receiving anthracycline-based therapy, trastuzumab, or combination regimens; (2) intervention with any beta-blocker (e.g., carvedilol, bisoprolol, metoprolol), either as monotherapy or used prophylactically as a cardioprotective agent; (3) comparator group receiving placebo, standard supportive care, or no beta-blocker therapy; (4) reporting outcomes related to LVEF (%), CTRCE, or providing sufficient data to calculate changes in LVEF and/or CTRCE. CTRCE was extracted according to each study's predefined criteria and generally included clinically significant LVEF decline and/or symptomatic heart failure. Some trials additionally reported arrhythmias or biomarker-defined myocardial injury as cardiotoxic events. Given variation in definitions across studies, CTRCE was analyzed as reported by the original investigators; compared with LVEF reduction, CTRCE represents a broader composite clinical endpoint; (5) study design restricted to RCTs.

Exclusion criteria were as follows: (1) duplicate publications or studies with overlapping patient populations, in which case the study with the largest sample size or most complete data was retained; (2) studies involving participants with pre-existing severe cardiac diseases (e.g., heart failure, myocardial infarction, or significant valvular disease); (3) interventions including additional cardioprotective agents (e.g., ACE inhibitors, ARBs, SGLT2 inhibitors) when data pertaining specifically to beta-blockers could not be extracted; (4) studies lacking relevant outcome measures or with missing or unobtainable data; (5) conference abstracts, case reports, review articles, and animal studies.

### 2.3 Literature screening

Reference management was conducted using EndNote X9.3.3 software (Clarivate Analytics, Philadelphia, PA, USA). Duplicate records were removed through a combination of automatic filtering and manual verification. Two reviewers independently screened titles and abstracts, followed by full-text assessment for potentially eligible studies. Disagreements were first resolved through discussion between the two reviewers. If consensus could not be reached, a third reviewer, who was not involved in the initial screening, independently reviewed the study and made the final decision. The literature selection process was visualized using a PRISMA flow diagram in accordance with PRISMA guidelines.

### 2.4 Data extraction

We designed and used a dedicated data extraction spreadsheet for this study. The extracted information included: (1) General study information: first author, publication year, sample size, mean age, and sex distribution of participants; (2) Study characteristics: intervention details, type of malignancy, and reported outcomes; (3) Methodological characteristics: randomization procedures, blinding methods, and other relevant design features. For continuous outcomes, we directly extracted change values (Δ) where reported. In cases where change values were not explicitly provided, we calculated the change by subtracting the baseline value from the endpoint value. For multi-arm trials, when the experimental groups had different doses, we pooled data across all experimental arms, disregarding dose variations. If the trials had different follow-up durations between arms, we selected data from the longest follow-up period for inclusion in the analysis. In cases of incomplete or insufficiently reported data, we attempted to contact the corresponding authors for clarification or additional information. Data extraction was conducted independently by two reviewers, and discrepancies were resolved through discussion or adjudication by a third reviewer.

### 2.5 Risk of bias assessment

The quality assessment of included studies was independently performed by two reviewers. The risk of bias for randomized controlled trials was evaluated using the Cochrane Risk-of-Bias Tool version 2 (RoB 2) (17). Disagreements between assessors regarding the RoB assessment were resolved through discussion between the two primary reviewers. In cases where consensus could not be reached, a third independent reviewer was consulted, and their judgment was used to finalize the assessment. However, due to the limited number of assessors and trials, a formal Kappa value or agreement rate was not calculated. The assessment covered five areas: (1) bias arising from the randomization process; (2) bias due to deviations from intended interventions; (3) bias due to missing outcome data; (4) bias in measurement of the outcome; (5) bias in selection of the reported result. Each included study was systematically evaluated and categorized as having “low risk,” “some concerns,” or “high risk” of bias based on the criteria outlined above.

### 2.6 Statistical analysis

The meta-analysis for this systematic review was conducted using STATA 16.0 and R 4.5.0 software. For LVEF, the primary effect measure was the mean change from baseline to the final follow-up. When change values were not directly reported, they were calculated from baseline and follow-up means where sufficient data were available. Weighted mean differences (WMD) with 95% confidence intervals were used to pool continuous outcomes, and dichotomous variables (such as CTRCE) were presented as relative risks (RR) with 95% confidence intervals (95% CI). In one multi-arm study, two experimental dose arms reported zero CTRCE events. As prespecified, experimental arms with different doses were pooled into a single intervention group. After pooling event counts and sample sizes, no zero-event comparisons remained in the final 2  ×  2 tables used for analysis. Therefore, continuity correction was not required. In traditional meta-analysis, the *I*^2^ statistic was used to assess heterogeneity between studies. When *I*^2^ < 50%, low heterogeneity was assumed, and a fixed-effects model was used for pooling; when *I*^2^ ≥ 50%, indicating significant heterogeneity, a random-effects model was applied. Subgroup analyses and meta-regression analysis were performed to explore potential sources of heterogeneity. Sensitivity analyses were conducted by sequentially excluding individual studies to verify the robustness of the results. Egger's test was used to detect publication bias. For the NMA, a Bayesian random-effects model was employed, and relative treatment effects were estimated using Markov Chain Monte Carlo (MCMC) simulation methods. Convergence of the MCMC chains was visually assessed using Gelman-Rubin statistics (R-hat), with the diagnostic plots provided in Supplementary Material S2. Model fit and consistency were evaluated using the Deviance Information Criterion (DIC). A difference of <5 between the consistency and inconsistency models was considered indicative of good model fit and overall consistency; a difference >5 suggested potential inconsistency. When closed loops were present within the evidence network, local consistency was further examined using node-splitting analysis. Based on model estimates, the surface under the cumulative ranking curve (SUCRA) was calculated to rank the cardioprotective efficacy of different beta-blockers. If the network lacked closed loops, only global consistency (based on DIC differences) was reported, and ranking was still performed using SUCRA.

## 3 Results

### 3.1 Literature screening process

**Figure 1:**
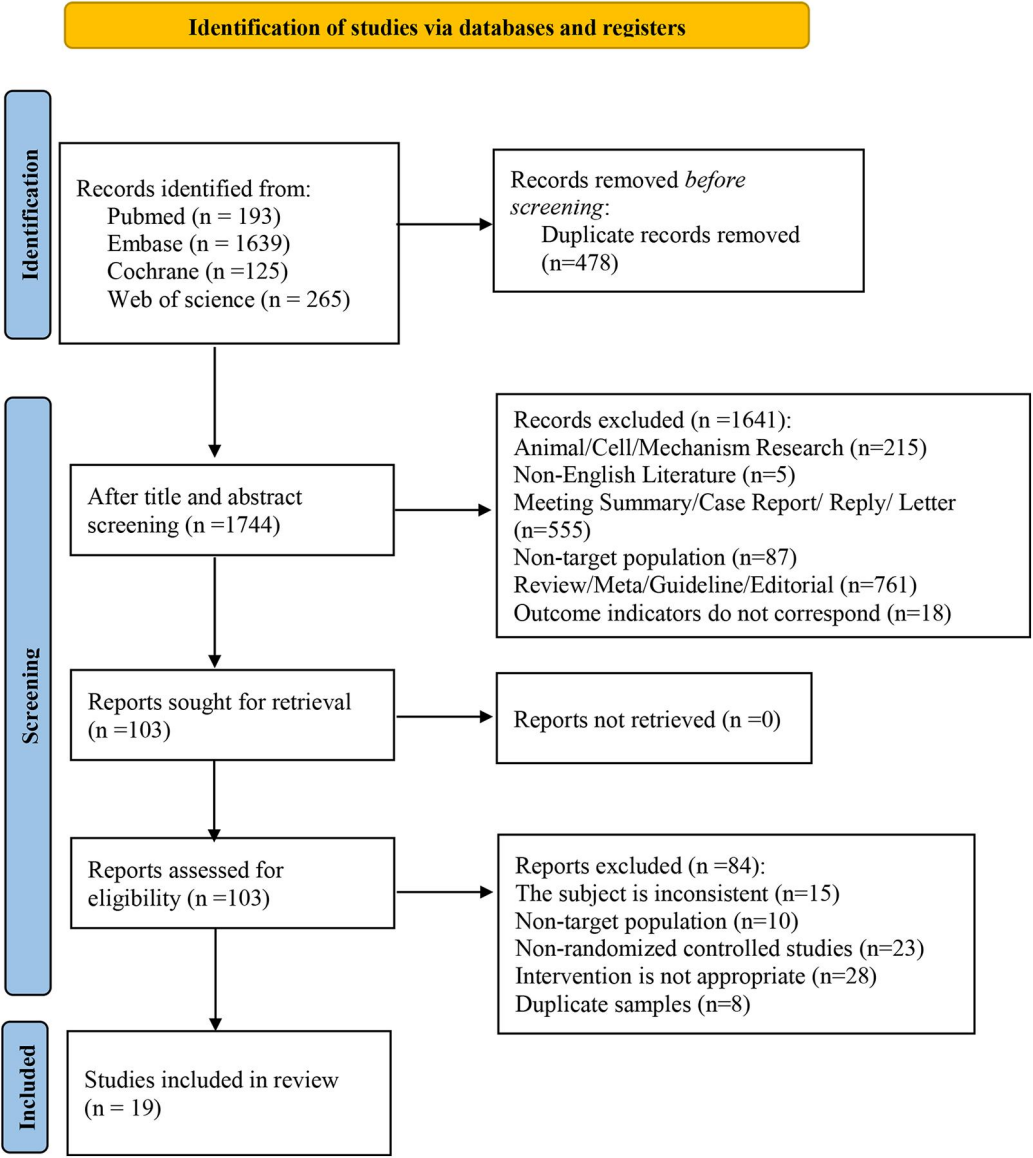
PRISMA literature screening flowchart.

A total of 2,222 records were retrieved from four databases. After removing 478 duplicates through both automated and manual review, 1,744 records remained for title and abstract screening. Of these, 1,641 studies were excluded for not meeting the inclusion criteria. The remaining 103 articles underwent full-text review, and 19 studies (14, 15, 18–27) met all eligibility criteria and were included in the final analysis. The overall study selection process is illustrated in Figure 1.

### 3.2 Basic characteristics of the included studies

Nineteen RCTs (11, 12, 14–15, 18–32) published between 2006 and 2025 were included, involving a total of 1,802 participants. Most studies enrolled breast cancer patients receiving anthracycline-based chemotherapy or trastuzumab, and the majority were single-center trials. Of the total sample, 177 were male and 1,625 were female. Geographically, 11 studies (12, 20–23, 25, 26, 28–31) were conducted in Asia, 5 (18, 20, 24, 29, 32) in Europe, and 3 (11, 14, 15) in the Americas. The BBs evaluated across the included trials were carvedilol, bisoprolol, metoprolol, and nebivolol. Detailed characteristics of each study are summarized in Table 1.

**Table 1:** Basic characteristics of the included studies.

Author	Country	Race	Sample Source	Disease	Chemotherapy regimen	Comparison	Samplesize(T/C)	Male/female	Age(Mean ± SD)	Outcomes
Abuosa, A. M. et al. (23)	Saudi Arabia	Asian	Single-center	Breast cancer/Lymphoma/Other	Anthracyclines	Carvedilol vs. Placebo	154 (116/38)	42/112	42.52 ± 15.30	LVEF/CTRCE
Pituskin, E. et al. (11)	Canada	American	Multi-center	Breast cancer	Trastuzumab	Bisoprolol vs. Placebo	61 (31/30)	0/61	52.02 ± 8.66	LVEF/CTRCE
Heck, S. L. et al. (27)	Norway	European	Single-center	Breast cancer	Anthracyclines/Trastuzumab	Metoprolol vs. Placebo	64 (32/32)	0/64	50.65 ± 9.15	LVEF/CTRCE
Kalay, N. et al. (12)	Turkey	Asian	Single-center	Breast cancer/Lymphoma/Other	Anthracyclines	Carvedilol vs. Placebo	50 (25/25)	7/43	47.90 ± 12.01	LVEF/CTRCE
Cochera, F. et al. (24)	Romania	European	Single-center	Breast cancer	Anthracyclines	Nebivolol vs. Placebo	60 (30/30)	0/60	52.60 ± 13.00	LVEF/CTRCE
Sherafati, A. et al. (30)	Iran	Asian	Single-center	Breast cancer	Trastuzumab	Carvedilol vs. Placebo	65 (27/38)	0/65	46.5 ± NR	LVEF
Avila, M. S. et al. (15)	Brazil	American	Single-center	Breast cancer	Anthracyclines	Carvedilol vs. Placebo	192 (96/96)	0/192	51.85 ± 9.62	LVEF/CTRCE
Tashakori Beheshti, A. et al. (31)	Iran	Asian	Single-center	Breast cancer	Anthracyclines	Carvedilol vs. Placebo	70 (30/40)	0/70	40.83 ± 6.27	LVEF
Kaya, M. G. et al. (28)	Turkey	Asian	Single-center	Breast cancer	Anthracyclines	Nebivolol vs. Placebo	45 (27/18)	0/45	51.05 ± 10.17	LVEF
Elitok, A. et al. (25)	Turkey	Asian	Single-center	Breast cancer	Anthracyclines	Carvedilol vs. Placebo	80 (40/40)	0/80	53.6 ± 10.24	LVEF
Nabati, M. et al. (29)	Iran	Asian	Single-center	Breast cancer	Anthracyclines	Carvedilol vs. Placebo	91 (46/45)	0/91	47.32 ± 10.60	LVEF/CTRCE
Meattini, I. et al. (32)	Florence	European	Multi-center	Breast cancer	Anthracyclines/Trastuzumab	Bisoprolol vs. Placebo	131 (66/65)	0/131	47.00 ± 10.50	LVEF/CTRCE
Esfandbod, M. et al. (26)	Iran	Asian	Single-center	Breast cancer	Anthracyclines/Trastuzumab	Carvedilol vs. Placebo	60 (30/30)	0/60	46.90 ± 9.30	LVEF/CTRCE
Stefanini, G. et al. (22)	Italy	European	Single-center	Breast cancer/Lymphoma	Anthracyclines	Nebivolol vs. Placebo	80 (40/40)	24/56	52.00 ± 14.00	LVEF/CTRCE
Salehi, R. et al. (21)	Iran	Asian	Single-center	Breast cancer/Lymphoma	Anthracyclines	Carvedilol vs. Placebo	66 (44/22)	20/46	46.67 ± 13.95	LVEF/CTRCE
Jhorawat R. et al. (19)	India	Asian	Single-center	Lymphoreticular malignancy	Anthracyclines	Carvedilol vs. Placebo	54 (27/27)	41/13	41.32 ± 2.35	LVEF/CTRCE
Guglin M. et al. (14)	America	American	Multi-center	Breast cancer	Anthracyclines/Trastuzumab	Carvedilol vs. Placebo	310 (156/154)	0/310	51.55 ± 0.60	LVEF/CTRCE
Livi L. et al. (20)	Italy	European	Multi-center	Breast cancer	Anthracyclines	Bisoprolol vs. Placebo	87 (45/42)	0/87	48.00 ± 12.75	LVEF/CTRCE
Georgakopoulos P. et al. (18)	Greece	European	Single-center	Lymphoma	Anthracyclines	Metoprolol vs. Placebo	82 (42/40)	43/39	50.07 ± 2.07	LVEF/CTRCE

### 3.3 Quality of the included studies

**Figure 2:**
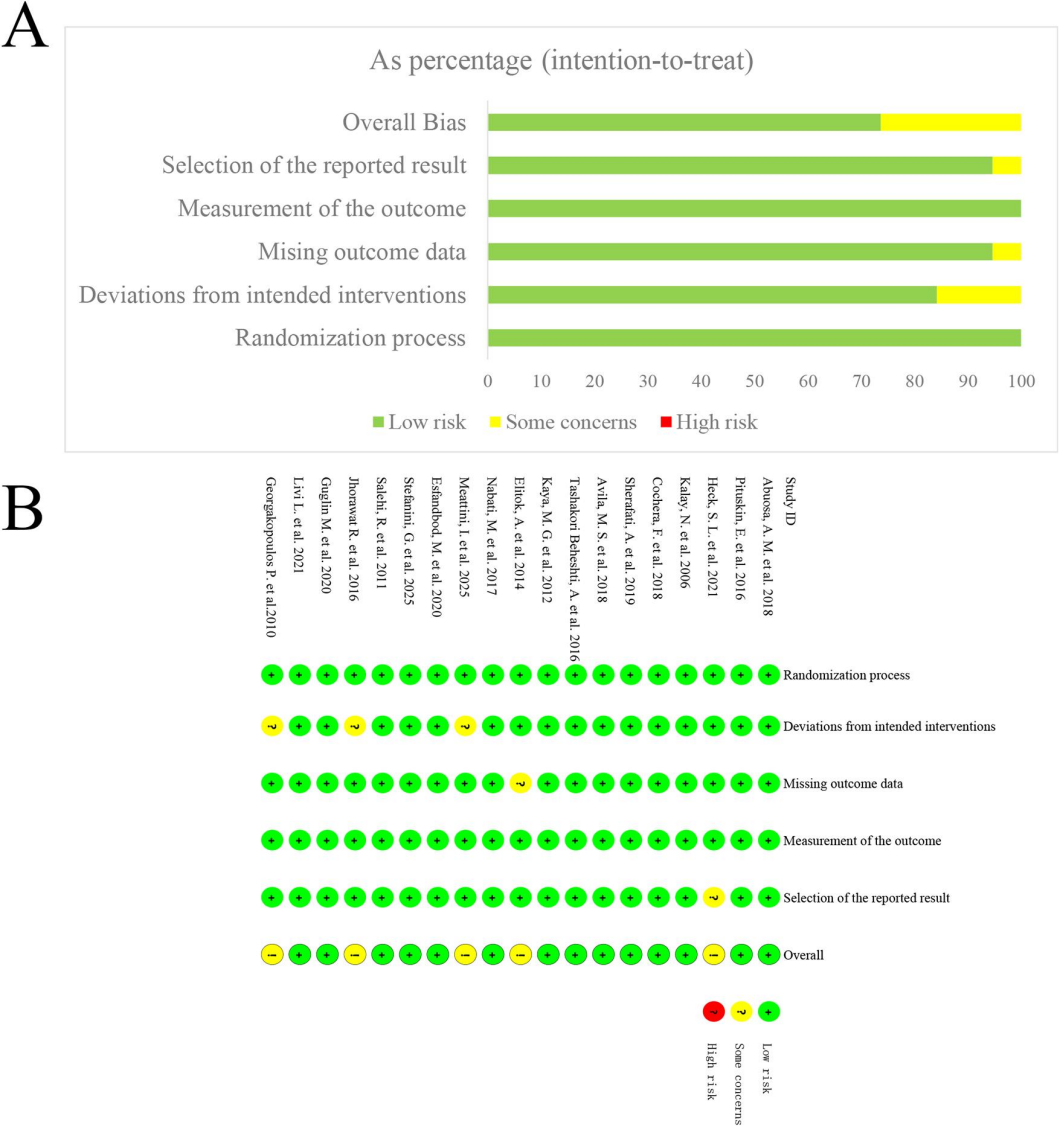
Risk of bias assessment of included RCTs: **(A)** domain-level summary; **(B)** overall traffic-light plot.

Based on the risk of bias assessment, all included studies showed low risk in terms of “randomization process” and “outcome measurement,” indicating that these studies had good methodological quality in terms of allocation and outcome measurement, ensuring the fairness and reliability of the results. However, a few studies raised potential concerns regarding “intervention bias” and “missing outcome data.” For instance, the studies by Meattini, I. et al. (32), Jhorawat, R. et al. (19), and Georgakopoulos, P. et al. (18) showed concerns regarding intervention bias, while the study by Elitok, A. et al. (25) had missing data that could potentially affect the reliability of the results. Regarding selective reporting, most studies presented complete reports, although the study by Heck, S. L. et al. (29) showed potential selective reporting bias, which could affect the comprehensiveness of the results. Overall, the 19 included RCTs were of moderate to high quality. The distribution of risk in the bias assessment is shown in Figure 2.

### 3.4 LVEF traditional meta-analysis results

**Figure 3:**
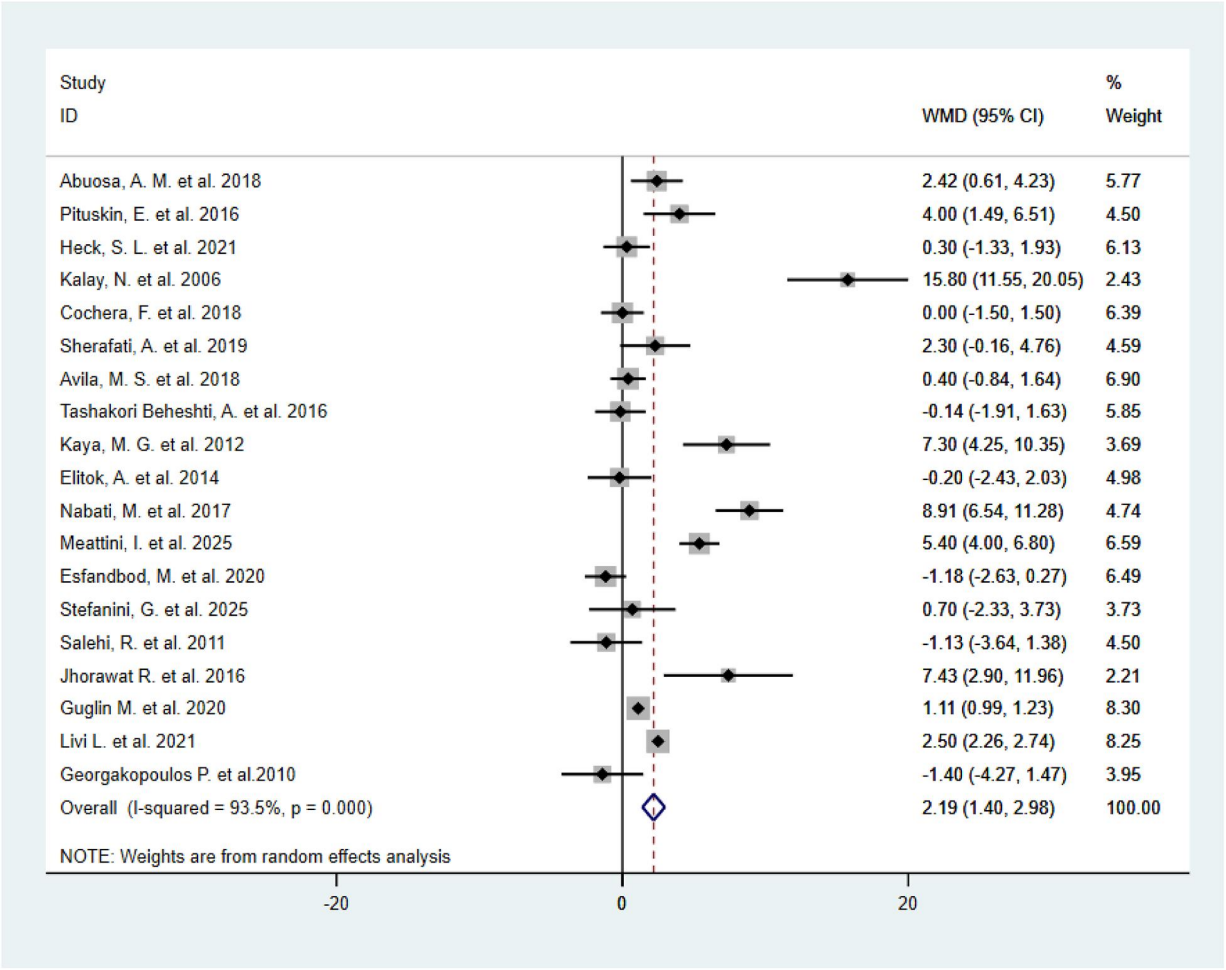
Forest plot of WMD in LVEF change between β-blockers and control groups (random-effects model, *n* = 1,802).

**Figure 4:**
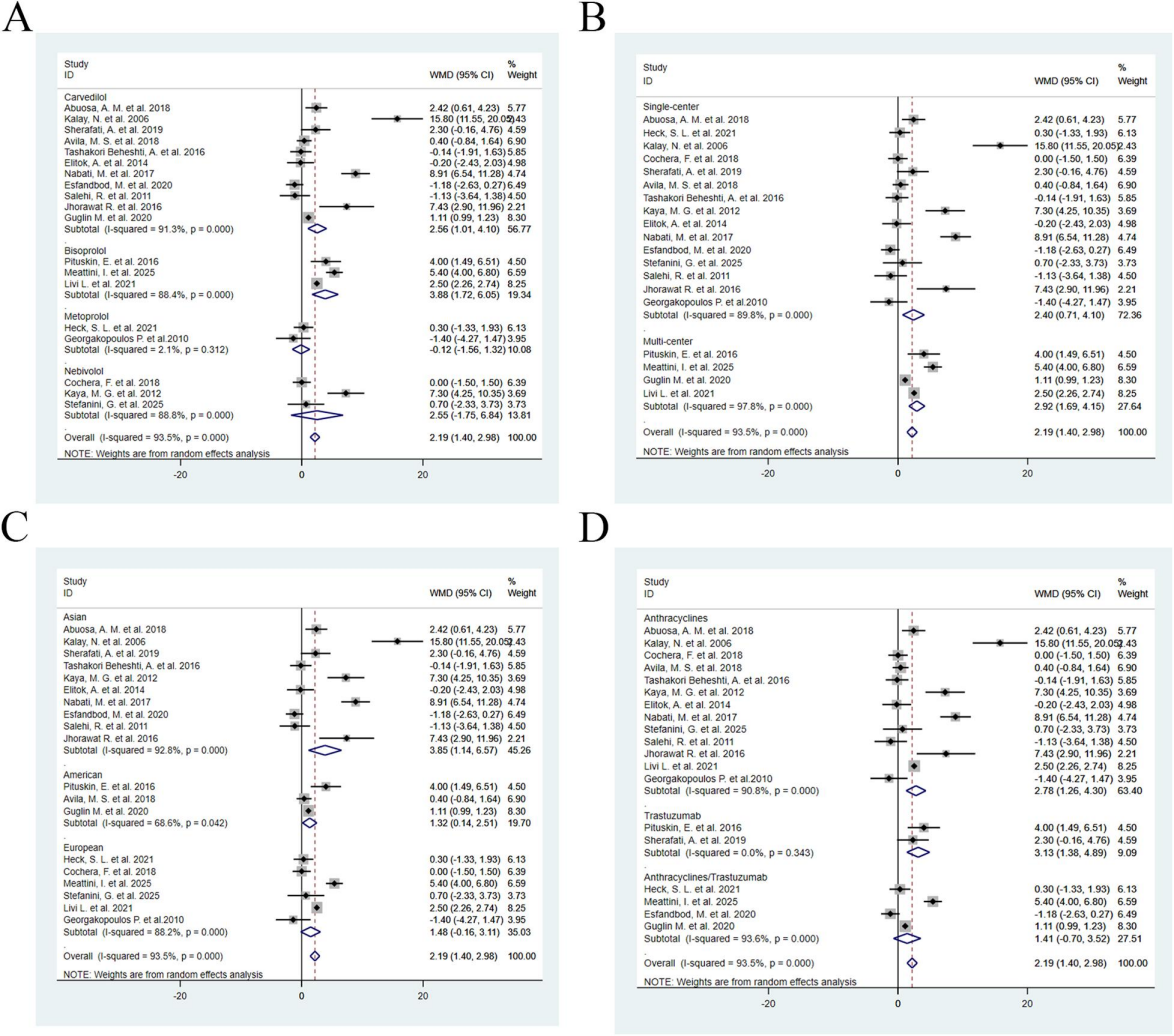
Subgroup analyses for LVEF: **(A)** by β-blocker (carvedilol 11 studies, *n* = 637; bisoprolol 3 studies, *n* = 142; metoprolol 2 studies, *n* = 74; nebivolol 3 studies, *n* = 97); **(B)** by study center (single-center 15 studies, *n* = 652; multi-center 4 studies, *n* = 298); **(C)** by race (Asian 10 studies, *n* = 412; American 3 studies, *n* = 283; European 6 studies, *n* = 255); **(D)** by chemotherapy regimen (anthracyclines 13 studies, *n* = 608; trastuzumab 2 studies, *n* = 58; anthracyclines/ trastuzumab 4 studies, *n* = 284).

A total of 19 randomized controlled trials (11, 12, 14, 15, 18–32) comparing beta-blockers with placebo were included in the analysis of LVEF outcomes. The overall combined results indicated a high degree of heterogeneity between studies (*I*^2^ = 93.5%), and a random-effects model was applied for analysis. The results showed that BBs significantly attenuated the decline in post-chemotherapy LVEF (WMD = 2.19, 95% CI: 1.40–2.98, *P* < 0.001), indicating a meaningful cardioprotective effect against anthracycline- and trastuzumab-associated left ventricular dysfunction (Figure 3). Sensitivity analyses demonstrated that sequential exclusion of individual studies did not materially alter the overall effect size, supporting the robustness of the findings (Supplementary Material S3). Egger's test suggested no significant publication bias (*P* = 0.25) (Supplementary Material S4). To further explore sources of heterogeneity, multiple subgroup analyses were conducted:

By Drug Type: The carvedilol group (12, 14, 15, 19, 21, 23, 25, 26, 29–31) (WMD = 2.56, 95% CI: 1.01–4.10, *P* = 0.001) and bisoprolol group (11, 20, 32) (WMD = 3.88, 95% CI: 1.72–6.05, *P* < 0.001) significantly improved LVEF, while the metoprolol group (18, 29) (WMD = −0.12, 95% CI: −1.56–1.32, *P* = 0.867) and nebivolol group (22, 24, 28) (WMD = 2.55, 95% CI: −1.75–6.84, *P* = 0.245) did not show statistical significance (Figure 4A).

By study center type: Both single-center studies (12, 15, 18, 19, 21–31) (WMD = 2.40, 95% CI: 0.71–4.10, *P* = 0.005) and multi-center studies (11, 14, 20, 32) (WMD = 2.92, 95% CI: 1.69–4.15, *P* < 0.001) observed a significant improvement in LVEF (Figure 4B).

By population subgroup: The Asian population (12, 20–23, 25, 26, 28–31) showed the most significant improvement (WMD = 3.85, 95% CI: 1.14–6.57, *P* = 0.005), followed by the American population (11, 14, 15) (WMD = 1.32, 95% CI: 0.14–2.51, *P* = 0.028), while the European population (18, 20, 24, 29, 32) (WMD = 1.48, 95% CI: −0.16–3.11, *P* = 0.077) did not show statistically significant results (Figure 4C).

By chemotherapy regimen: Anthracycline therapy (12, 15, 18–25, 27–29) (WMD = 2.78, 95% CI: 1.26–4.30, *P* < 0.001) and trastuzumab therapy (11, 30) (WMD = 3.13, 95% CI: 1.38–4.89, *P* < 0.001) significantly improved LVEF, whereas combined anthracycline/trastuzumab therapy (14, 26, 29, 32) (WMD = 1.41, 95% CI: −0.70–3.52, *P* = 0.189) did not show a significant effect (Figure 4D).

To explore the sources of high heterogeneity (*I*^2^ = 93.5%) in the LVEF analysis, we conducted a meta-regression considering baseline LVEF as a potential covariate. The meta-regression results indicated that baseline LVEF was not a significant predictor of the treatment effect (Supplementary Material S5).

### 3.5 Bayesian network meta-analysis results for LVEF

**Figure 5:**
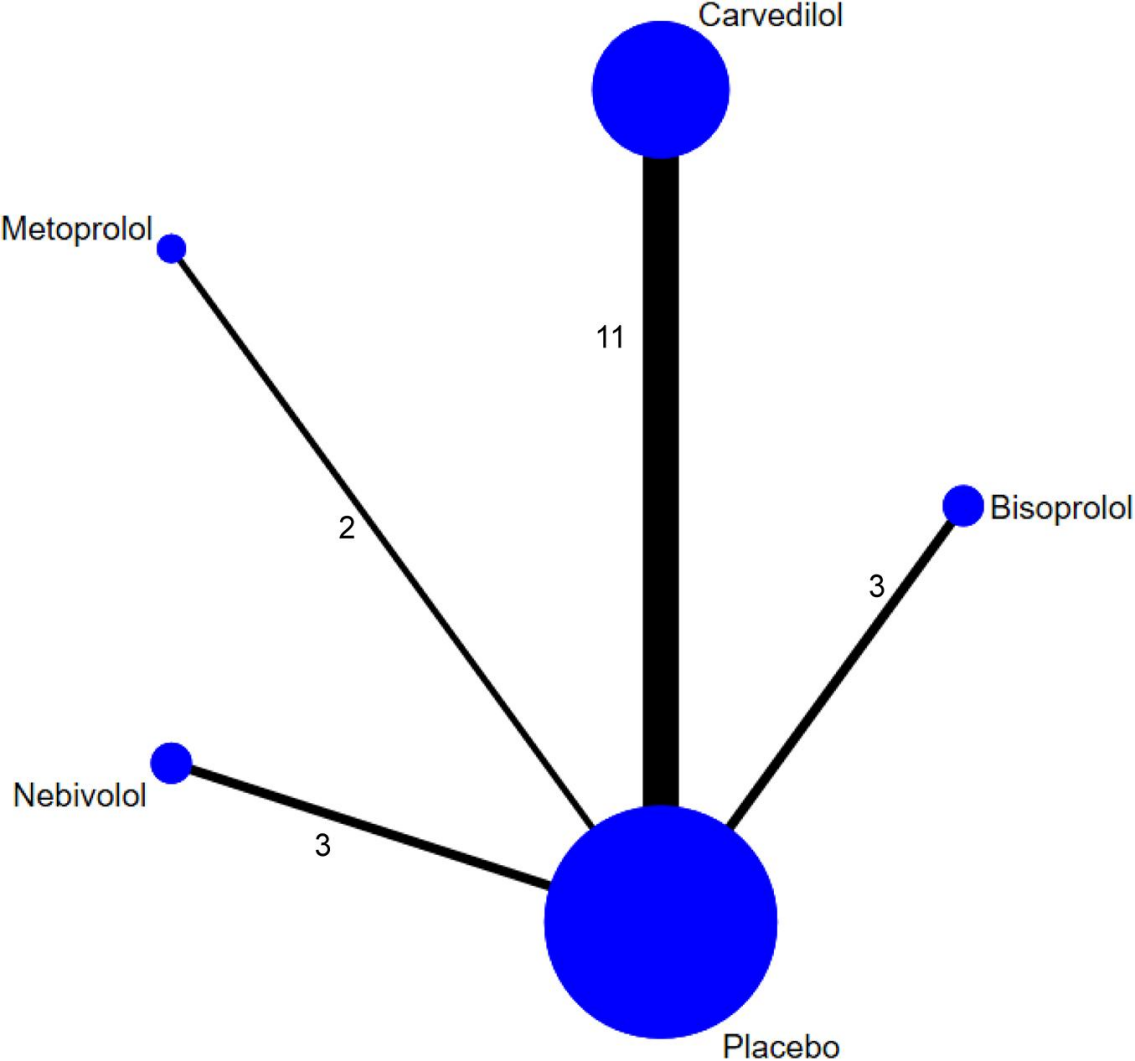
Network plot of treatment comparisons for LVEF (NMA). Node size represents the sample size for each treatment group. The number of studies for each direct comparison is indicated by the number next to the lines connecting the nodes.

To further explore differences in the efficacy of various BBs, we conducted a network meta-analysis (NMA). A total of 19 RCTs (11, 12, 14, 15, 18–32) were included. The network diagram (Figure 5) illustrates the distribution of interventions and sample sizes. Model fit analysis revealed that the deviance information criterion (DIC) difference between the consistency and inconsistency models was less than 5 (Supplementary Material S6), indicating comparable model performance; therefore, the consistency model was used for reporting final results.

**Figure 6:**
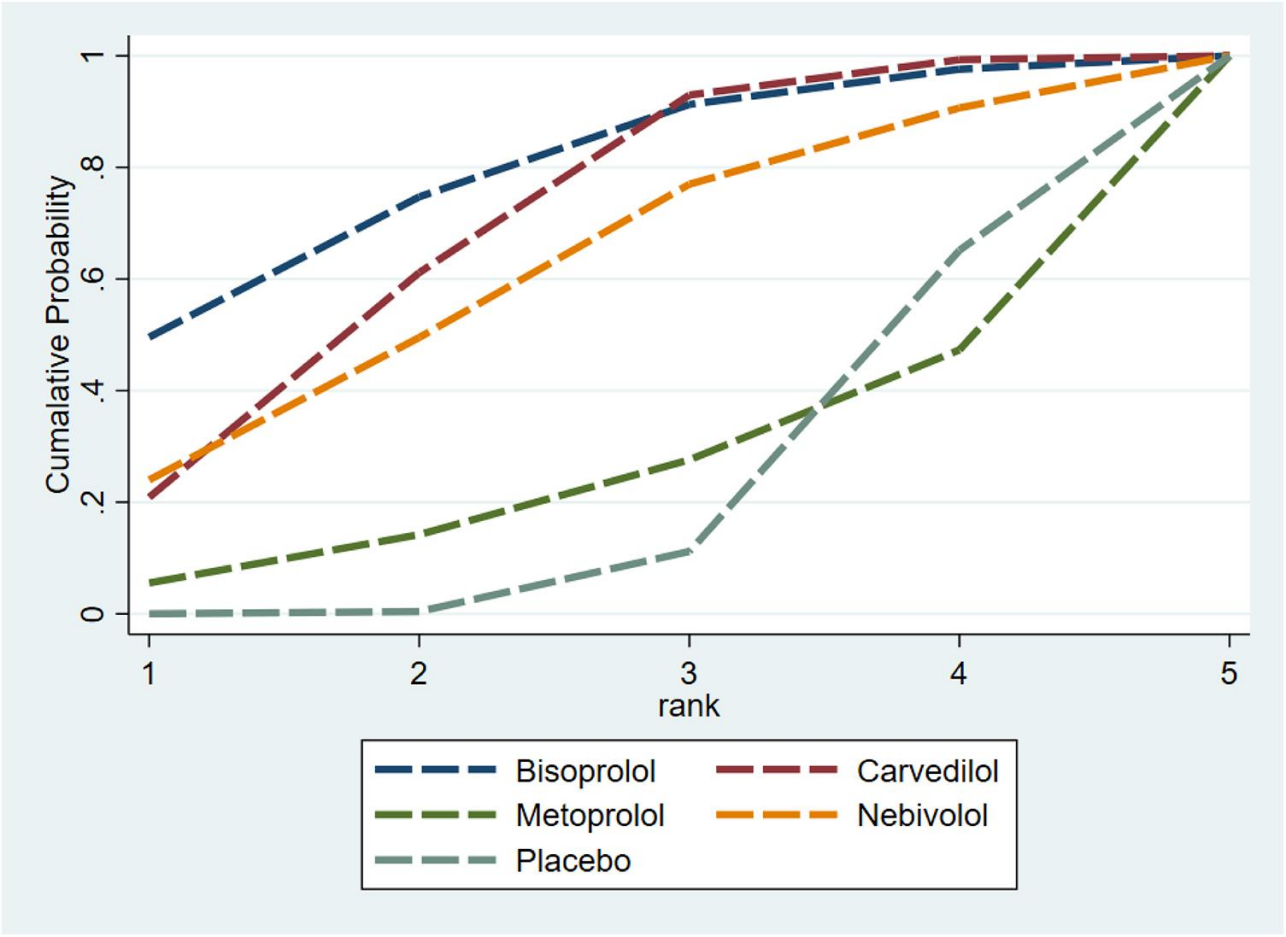
SUCRA rankings of LVEF protection across β-blockers. Bisoprolol: SUCRA = 0.7696; Carvedilol: SUCRA = 0.6822; Nebivolol: SUCRA = 0.6023; Metoprolol: SUCRA = 0.2413; Placebo: SUCRA = 0.2047.

The SUCRA results showed that Bisoprolol (SUCRA = 0.7696) performed best in improving LVEF, followed by Carvedilol (SUCRA = 0.6822), Nebivolol (SUCRA = 0.6023), and Metoprolol (SUCRA = 0.2413), with Placebo (SUCRA = 0.2047) performing the worst (Figure 6). The funnel plot was nearly symmetric, indicating no publication bias (Supplementary material S7).

### 3.6 Traditional meta-analysis results for CTRCE

**Figure 7:**
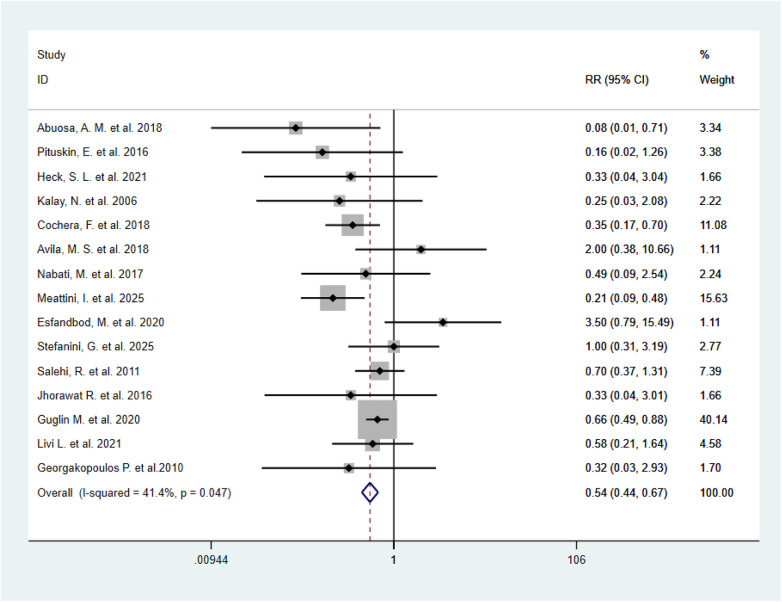
Forest plot of RR for CTRCE, comparing β-blockers vs. control (*n* = 1,542).

A total of 15 RCTs (11, 12, 14, 15, 18–24, 26, 27, 29, 32) were included to evaluate the effect of BBs vs. placebo on the incidence of CTRCE. The overall pooled results indicated acceptable heterogeneity (*I*^2^ = 41.4%), and the fixed-effect model was applied. The results showed that BBs significantly reduced the incidence of CTRCE (RR = 0.54, 95% CI: 0.44–0.67, *P* < 0.001), suggesting that BBs effectively protect against anthracycline- and trastuzumab-related CTRCE (Figure 7). Sensitivity analysis showed that sequential removal of any single study did not materially alter the overall effect size, indicating robust findings (Supplementary Material S8). Egger's test revealed no evidence of publication bias (*P* = 0.39) (Supplementary Material S9). To further explore potential sources of heterogeneity, multiple subgroup analyses were conducted.

**Figure 8:**
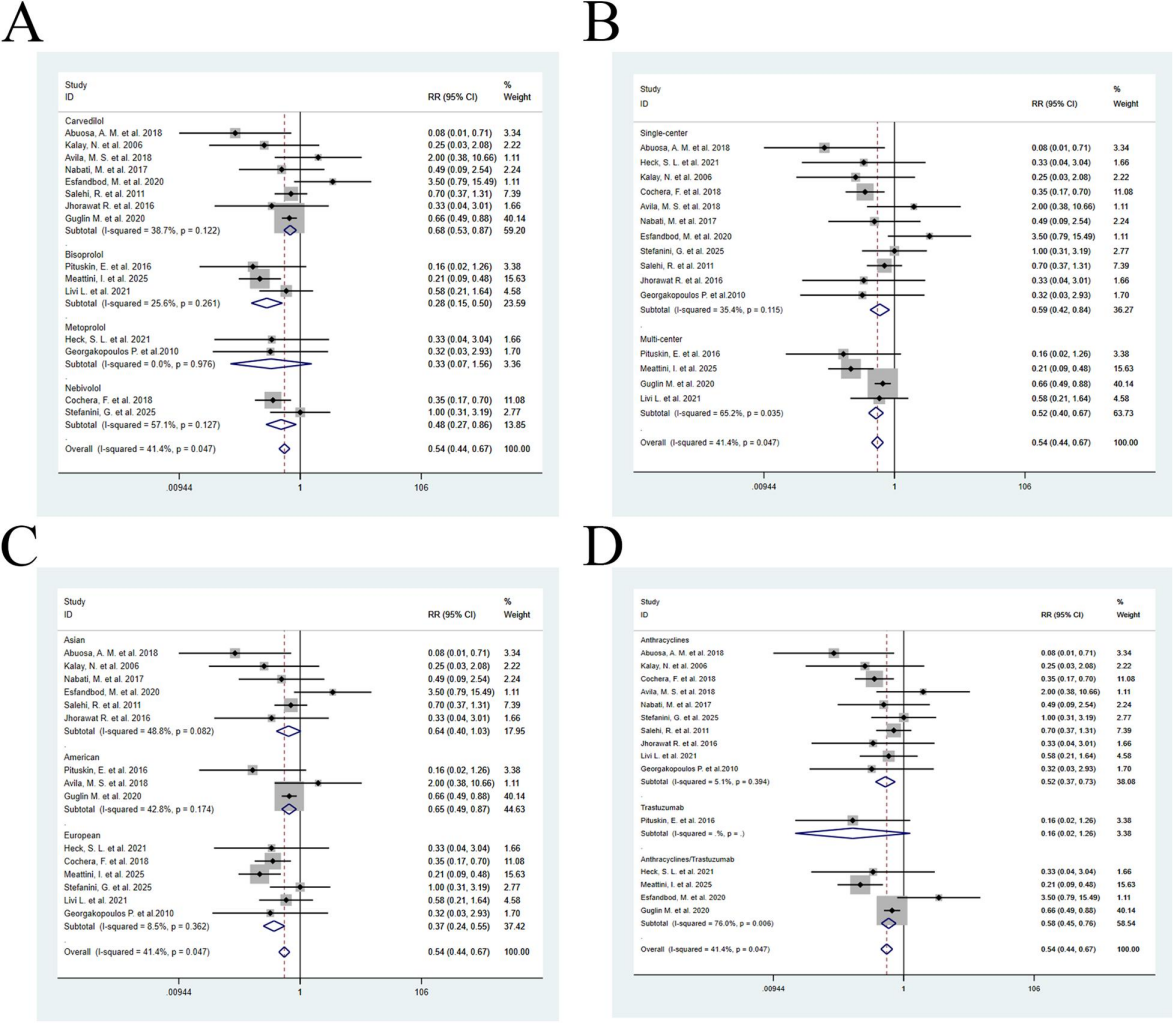
Subgroup analyses for CTRCE incidence. **(A)** by β-blocker (Carvedilol 8 studies, *n* = 540; Bisoprolol 3 studies, *n* = 142; Metoprolol 2 studies, *n* = 74; Nebivolol 2 studies, *n* = 70); **(B)** by study center (Single-center 11 studies, *n* = 528; Multi-center 4 studies, *n* = 298); **(C)** by race (Asian 6 studies, *n* = 288; American 3 studies, *n* = 283; European 6 studies, *n* = 255); **(D)** by chemotherapy regimen (Anthracyclines 10 studies, *n* = 511; Trastuzumab 1 studies, *n* = 31; Anthracyclines/ Trastuzumab 4 studies, *n* = 284).

By drug type: The Carvedilol group (12, 14, 15, 19, 21, 23, 25, 26, 29–31) (RR = 0.68, 95% CI: 0.53–0.87, *P* = 0.002), Bisoprolol group (11, 20, 32) (RR = 0.28, 95% CI: 0.15–0.50, *P* < 0.001), and Nebivolol group (22, 24, 28) (RR = 0.48, 95% CI: 0.27–0.86, *P* = 0.013) all significantly reduced the incidence of CTRCE, whereas the Metoprolol group (18, 29) (RR = 0.33, 95% CI: 0.07–1.56, *P* = 0.160) did not show statistical significance (Figure 8A).

By study center type: Both single-center studies (12, 15, 18, 19, 21–31) (RR = 0.59, 95% CI: 0.42–0.84, *P* = 0.003) and multi-center studies (11, 14, 20, 32) (RR = 0.52, 95% CI: 0.40–0.67, *P* < 0.001) observed a significant reduction in the incidence of CTRCE (Figure 8B).

By population subgroup: The American population (11, 14, 15) (RR = 0.65, 95% CI: 0.49–0.87, *P* = 0.003) and the European population (18, 20, 24, 29, 32) (RR = 0.37, 95% CI: 0.24–0.55, *P* < 0.001) both showed a significant reduction in CTRCE incidence, whereas the Asian population (12, 20–23, 25, 26, 28–31) (RR = 0.64, 95% CI: 0.40–1.03, *P* = 0.064) did not show statistical significance (Figure 8C).

By chemotherapy regimen: Anthracycline therapy (12, 15, 18–25, 27–29) (RR = 0.52, 95% CI: 0.37–0.74, *P* < 0.001) and combined anthracycline/trastuzumab therapy (14, 26, 29, 32) (RR = 0.58, 95% CI: 0.45–0.79, *P* < 0.001) significantly reduced the incidence of CTRCE, while trastuzumab therapy alone (11, 30) (RR = 0.16, 95% CI: 0.02–1.26, *P* = 0.082) did not show a significant effect (Figure 8D).

### 3.7 Bayesian network meta-analysis results for CTRCE

**Figure 9:**
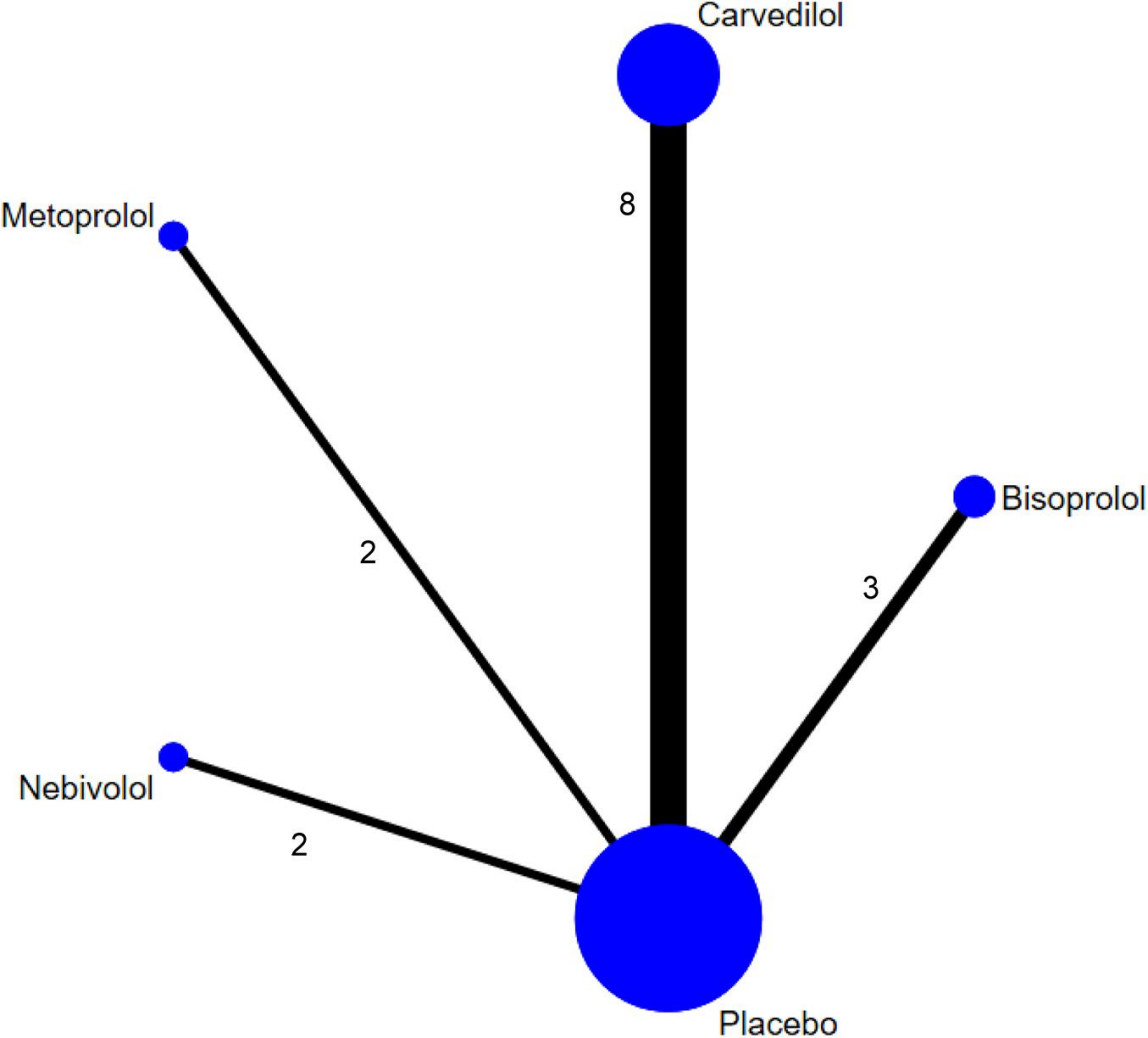
Network plot of treatment comparisons for CTRCE (NMA). Node size represents the sample size for each treatment group. The number of studies for each direct comparison is indicated by the number next to the lines connecting the nodes.

**Figure 10:**
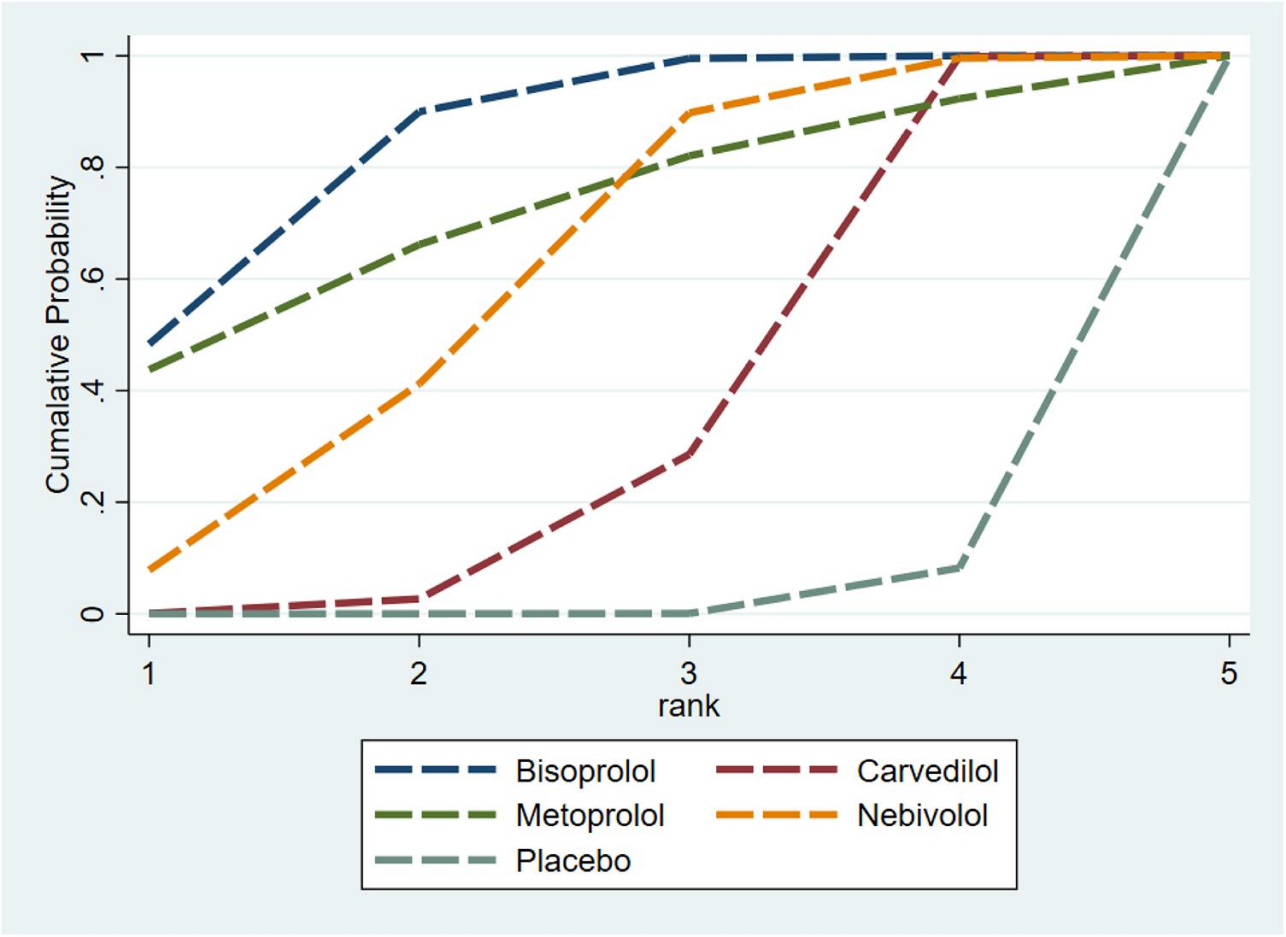
SUCRA rankings for CTRCE incidence. Bisoprolol: SUCRA = 0.8085; Metoprolol: SUCRA = 0.7303; Nebivolol: SUCRA = 0.5124; Carvedilol: SUCRA = 0.3743; Placebo: SUCRA = 0.0745.

A total of 15 RCTs (11, 12, 14, 15, 18–24, 26, 27, 29, 32) were included in the NMA (Figure 9). Model fit assessment showed that the DIC difference between the consistency and inconsistency models was less than 5 (Supplementary Material S10), indicating comparable fit quality; therefore, the consistency model was adopted for final reporting. According to SUCRA rankings, Bisoprolol (SUCRA = 0.8085) showed the strongest effect in reducing CTRCE incidence, followed by Metoprolol (0.7303), Nebivolol (0.5124), and Carvedilol (0.3743), with Placebo ranked last (0.0745) (Figure 10). The funnel plot appeared symmetrical, suggesting no publication bias (Supplementary Material S11).

## 4 Discussion

This study systematically integrated evidence from RCTs using both traditional meta-analysis and Bayesian NMA to evaluate the role of beta-blockers (BBs) in preventing cardiotoxicity induced by anthracyclines and trastuzumab. The findings support BBs as a cardioprotective strategy in chemotherapy-related cardiac injury. BBs significantly improved post-chemotherapy LVEF and reduced CTRCE incidence, with bisoprolol showing the most pronounced effects in both LVEF improvement and cardiac protection. Previous studies have suggested that BBs may help mitigate chemotherapy-related cardiotoxicity, but these studies were limited to single agents or lacked direct comparisons, and their generalizability was constrained by variations in populations and outcomes (33–35). By including multiple BBs and performing NMA-based indirect comparisons, this study systematically compared their relative efficacy. Bisoprolol and carvedilol ranked highest in SUCRA values, suggesting favorable performance in LVEF preservation, although statistical uncertainty remains. Carvedilol, for example, has been shown to exert antioxidant effects by reducing reactive oxygen species (ROS), a key factor in chemotherapy-induced myocardial damage. Studies have demonstrated that carvedilol helps reduce myocardial apoptosis by inhibiting ROS formation, which improves myocardial cell survival during chemotherapy (8, 36). Similarly, bisoprolol, a β₁-selective blocker, enhances myocardial oxygenation and reduces oxidative stress, which is particularly beneficial in preventing chemotherapy-induced cardiotoxicity (37). These mechanisms likely explain the greater efficacy of bisoprolol and carvedilol in preserving LVEF compared to metoprolol and nebivolol, which lack these additional protective pathways.

In contrast, metoprolol and nebivolol showed more limited effects, possibly due to the absence of antioxidant and anti-apoptotic mechanisms (18, 35). Metoprolol, as a β₁-selective blocker, primarily acts on the myocardium, but its lack of significant antioxidant and anti-apoptotic actions may limit its cardioprotective effects in chemotherapy-induced cardiotoxicity. Unlike bisoprolol and carvedilol, which actively combat oxidative stress, metoprolol's mechanisms focus primarily on reducing heart rate and myocardial oxygen demand, which may not be sufficient to counteract the oxidative damage caused by chemotherapy agents (38). Similarly, nebivolol, despite some antioxidant effects via nitric oxide-mediated pathways, lacks the strong anti-apoptotic properties that are seen in carvedilol (39). This difference in their pharmacological profiles likely explains why nebivolol, like metoprolol, has more limited efficacy in preserving LVEF and reducing chemotherapy-induced myocardial injury.

It also explored the role of BBs in protecting against clinical heart events. The results showed that BBs significantly reduced the incidence of CTRCE, further validating their important role in improving LVEF and emphasizing their real-world protective effects on clinical heart events. Specifically, BBs were found to reduce the risks of heart failure, arrhythmias, and acute myocardial injury, indicating their protective effects extend beyond just preserving cardiac function. Bisoprolol ranked highest in reducing CTRCE incidence, although the evidence for some comparisons was based on a limited number of trials. And followed by Metoprolol and Nebivolol, which further reinforced the clinical value of BBs in protecting against chemotherapy-related cardiotoxicity. The CTRCE results also showed that BBs effectively reduced the risks of heart failure, arrhythmias, and acute myocardial injury, indicating that their protective effects extend beyond cardiac function to include clinical endpoints. Importantly, different BBs demonstrated consistent trends in both functional and clinical outcomes, indicating a certain inherent coherence in their protective mechanisms.

Additionally, we explored the efficacy differences of BBs through subgroup analyses based on different ethnicities, chemotherapy regimens, and types of research centers, thereby further deepening our understanding of the drug's therapeutic diversity. The results showed that both the Asian and American populations demonstrated more significant improvements in LVEF, while statistically significant differences in the reduction of CTRCE incidence were observed between the American and European populations. This phenomenon may reflect differences in drug metabolism rates, receptor expression, and genetic polymorphisms across populations. Although no systematic studies have previously focused on the efficacy differences among different populations for this indication, existing literature suggests that racial/ethnic differences may influence the incidence and recovery of chemotherapy-related cardiotoxicity (40). Other studies have also indicated that genetic polymorphisms can affect susceptibility to anthracycline-induced cardiotoxicity (41). Whether in single-center or multi-center studies, BBs consistently demonstrated protective effects in improving LVEF and reducing CTRCE, suggesting that its efficacy is robust and reliable.

It is noteworthy that while BBs can improve LVEF and reduce CTRCE in anthracycline-induced cardiac injury, the trends for these two outcomes show some differences in the context of trastuzumab or combination chemotherapy regimens. Specifically, only one study was included in the CTRCE analysis for trastuzumab-induced cardiotoxicity, and the sample size was small, leading to weaker evidence strength, so this result should not be over-interpreted. In patients treated with both anthracyclines and trastuzumab, BBs did not significantly improve LVEF, but were still able to further reduce CTRCE. This difference may be related to the multiple cardiac toxicity mechanisms induced by the synergistic effects of the two drugs. Anthracyclines induce oxidative stress and mitochondrial damage, leading to myocardial cell necrosis, while trastuzumab interferes with myocardial repair by inhibiting the HER2 signaling pathway (42, 43). The combination of these two factors may place the myocardium in a state of continuous damage, thereby diminishing the protective effect of BBs as a monotherapy. Therefore, future studies should explore more individualized and multi-targeted intervention strategies, such as combining BBs with ACE inhibitors or SGLT2 inhibitors, to enhance the overall protective effect against chemotherapy-related cardiac injury.

This study further demonstrates that bisoprolol and carvedilol show significant efficacy in preventing chemotherapy-related cardiac dysfunction. In addition to their classical β₁-receptor–blocking effects, both medications possess antioxidant, anti-apoptotic, and endothelial function–improving properties, enabling them to mitigate chemotherapy-induced myocardial injury through multiple pathways. Specifically, bisoprolol, based on its β₁-selective blockade, can enhance cardiac perfusion and oxygenation, thereby providing stable cardioprotective effects (42). Carvedilol, a non-selective β–*α* receptor antagonist, further reduces cardiac preload and afterload as well as oxygen demand, while exerting antioxidant and anti-apoptotic effects, thus enhancing cardiac tolerance during chemotherapy (44). In contrast, although Metoprolol and Nebivolol also offer some protective effects, they did not achieve statistical significance in the meta-analysis, which may be related to differences in their pharmacological mechanisms. Metoprolol, as a β₁-selective blocker, primarily acts on the myocardium but lacks substantial antioxidant and anti-apoptotic capabilities (18), consistent with previous findings (45). Although Nebivolol can generate some antioxidant effects through NO-mediated pathways, its mild pharmacological effect may be insufficient to provide significant protection against the cardiac toxicity induced by high-dose chemotherapy agents (35). Therefore, despite their potential clinical relevance, both agents may have limited myocardial protective capacity in the setting of complex chemotherapy regimens.

In the CTRCE analysis, the advantage of bisoprolol was reaffirmed, showing the strongest protective effect, followed by metoprolol, nebivolol, and carvedilol. However, unlike the results of the traditional meta-analysis, metoprolol did not achieve statistical significance in pairwise comparisons but ranked favorably in the NMA. This is mainly due to the network meta-analysis integrates both direct and indirect evidence, enhancing the overall probability-based ranking of relative effects. This reflects the relative stability across studies and comparisons, and does not imply that its absolute effect is stronger than that of Bisoprolol or Carvedilol.

As with any network meta-analysis, the validity of indirect comparisons relies on the assumption of transitivity. The included trials differed in cancer type, chemotherapy regimen, baseline cardiovascular risk, and follow-up duration. We therefore considered whether these potential effect modifiers were reasonably balanced across treatment comparisons. Although clinical variability was present, no clear systematic imbalance between specific beta-blocker comparisons was identified. Nevertheless, given the heterogeneity inherent to oncology populations and treatment protocols, residual confounding cannot be excluded. Accordingly, the SUCRA-based rankings should be interpreted cautiously and regarded as comparative rather than definitive evidence of superiority. It should also be noted that several treatment nodes were informed by a relatively small number of trials, and credible intervals for some indirect comparisons were wide. Therefore, although SUCRA rankings provide useful comparative insights, they should be interpreted within the context of statistical uncertainty and the underlying evidence base.

Despite the overall consistency of the findings, the LVEF analysis was characterized by substantial heterogeneity (*I*^2^ ≈ 93%), which inevitably limits the interpretability of a single pooled estimate. Although differences in beta-blocker dosage and intervention duration may partly explain this variability, other clinically relevant factors likely contributed as well. Baseline LVEF and underlying cardiac reserve differed across trials, and cumulative anthracycline exposure varied considerably, both of which are known to influence the risk and magnitude of cardiotoxicity. In addition, studies used different imaging modalities and follow-up time points to assess LVEF, and initiation timing and treatment duration of beta-blockers were not uniform. These variations may have meaningfully affected the observed treatment effects. Geographic differences may also have played a role, as patients from different regions may vary in drug metabolism, receptor sensitivity, and chemotherapy intensity. For example, polymorphisms in β-receptors and drug-metabolizing enzymes have been reported to differ across populations (46, 47), potentially influencing responsiveness to cardioprotective therapy. Because these variables were inconsistently reported, further stratified analyses were not feasible. Therefore, the pooled LVEF estimate should be interpreted cautiously. Future studies with more standardized reporting and longer follow-up are needed to clarify these sources of variability and improve precision in treatment selection.

In summary, the CTRCE analysis indicates that the protective effects of BBs extend beyond improvements in cardiac function to clinically meaningful events. Bisoprolol consistently ranked highest across outcomes; however, these rankings reflect relative probabilities and should not be interpreted as definitive evidence of superiority. BBs maintained stable efficacy across different centers and chemotherapy regimens, supporting their value for broader clinical application. Future research should further validate their long-term protective effects across diverse populations and chemotherapy protocols, and explore combined interventions or early preventive strategies to optimize cardioprotection in chemotherapy patients. Despite providing supportive evidence for the use of beta-blockers (BBs) in chemotherapy-related cardiotoxicity, this study has several limitations. Significant variations in the dosage, initiation timing, and treatment duration of beta-blockers across the included studies need to be acknowledged. These factors could have substantially influenced the observed efficacy, as the pharmacokinetics and pharmacodynamics of each drug can vary depending on dosage and duration. Furthermore, differences in follow-up duration across trials may have contributed to heterogeneity in the results. First, the follow-up duration in the included trials was generally short, which limits the ability to assess the long-term efficacy and safety of beta-blocker treatment. Second, some subgroups (e.g., trastuzumab monotherapy) were based on only 1–3 studies, which restricts statistical power and generalizability. Third, a few studies had small sample sizes or incomplete data reporting, which could affect the overall representativeness of the results. Additionally, although formal tests did not indicate significant publication bias, the relatively small number of trials for certain outcomes reduces the power of statistical assessments for small-study effects. Therefore, the possibility that smaller studies with larger effect sizes may have influenced the pooled estimates, particularly for left ventricular ejection fraction (LVEF), cannot be entirely excluded. Another limitation is the number of treatment nodes within the network meta-analysis, which were supported by a limited number of trials. Indirect comparisons, in some cases, were associated with wide credible intervals, indicating statistical uncertainty. Furthermore, the definition of chemotherapy-related cardiotoxicity events (CTRCE) across the trials varied. Although analyzed as a composite endpoint, the components differed between studies, encompassing events of varying clinical severity, such as asymptomatic LVEF decline, overt heart failure, arrhythmias, and biomarker-defined myocardial injury. These variations could affect comparability and should be considered when interpreting the pooled estimates. Moreover, the incomplete reporting of drug dosage and follow-up duration across the included studies presents another limitation. These factors are crucial in determining the efficacy of beta-blockers; however, due to limited or inconsistent reporting, we were unable to fully assess their potential impact on treatment outcomes. This lack of comprehensive data reduces the ability to explore sources of heterogeneity in the meta-regression analysis. Finally, the search strategy was confined to major bibliographic databases, and we did not systematically search grey literature or clinical trial registries, which may have led to the omission of unpublished or ongoing trials. Future research should incorporate larger, well-designed randomized controlled trials (RCTs) with standardized outcome definitions, longer follow-up periods, more comprehensive literature searches, and more complete data reporting to improve the robustness and clinical applicability of the findings.

## 5 Conclusion

This study systematically integrated evidence from multiple RCTs and suggests that beta-blockers may provide functional and clinical benefits in the prevention of chemotherapy-related cardiotoxicity. Overall, BBs were associated with preservation of LVEF and a reduction in CTRCE incidence in patients receiving anthracycline- and trastuzumab-based therapies. Among the evaluated agents, bisoprolol and carvedilol ranked more favorably across outcomes; however, these findings reflect relative probabilities within the network and should not be interpreted as definitive evidence of superiority. Given the heterogeneity of included trials and the limited number of studies informing some treatment comparisons, the current evidence should be viewed as supportive but not conclusive. Future large-scale, well-designed RCTs with longer follow-up are warranted to further clarify comparative efficacy and inform clinical decision-making.

## Data Availability

The original contributions presented in the study are included in the article/Supplementary Material, further inquiries can be directed to the corresponding author.

## Supplementary material

Supplementary Materials S1 Search History.

Supplementary Material S2 Details of Network Meta-Analysis.

Supplementary Material S3 Sensitivity analysis of the effect of β-blockers on LVEF improvement.

Supplementary Material S4 Egger's test for publication bias in traditional meta-analysis of LVEF.

Supplementary Material S5 Meta-regression Analysis for LVEF Change.

Supplementary Material S6 DIC results for LVEF network meta-analysis.

Supplementary Material S7 Funnel plot for LVEF network meta-analysis.

Supplementary Material S8 Sensitivity analysis for CTRCE incidence.

Supplementary Material S9 Egger's test for publication bias in traditional meta-analysis of CTRCE.

Supplementary Material S10 DIC results for CTRCE network meta-analysis.

Supplementary Material S11 Funnel plot for CTRCE network meta-analysis.
