# Supplementary material for: Protective effects of beta-blockers against anthracycline- and trastuzumab-related cardiotoxicity: a systematic review based on conventional and Bayesian network meta-analysis
Source: Front Cardiovasc Med. 2026 Apr 1;13:1777908. doi: 10.3389/fcvm.2026.1777908 (PMC13079159; doi:10.3389/fcvm.2026.1777908)
Supplement: Supplementary Materials S1 — Search History. [file Table1.docx]

**Pubmed**

| Search number | Query | Search Details | Results |
| --- | --- | --- | --- |
| 1 | ((((((((((((((((((((((((Adrenergic beta-Antagonists[MeSH Terms]) OR (Adrenergic beta Antagonist*[Title/Abstract])) OR (Adrenergic beta Receptor Blockader*[Title/Abstract])) OR (beta Adrenergic Antagonist*[Title/Abstract])) OR (beta Adrenoceptor Antagonist*[Title/Abstract])) OR (beta Adrenergic Blocking Agent*[Title/Abstract])) OR (beta Adrenergic Receptor Blockader*[Title/Abstract])) OR (beta Adrenergic Blocker*[Title/Abstract])) OR (beta adrenoceptor blocking drug[Title/Abstract])) OR (beta adrenolytic[Title/Abstract])) OR (beta adrenolytic agent[Title/Abstract])) OR (beta blocking adrenergic agent[Title/Abstract])) OR (beta blocking agent[Title/Abstract])) OR (beta blocking drug[Title/Abstract])) OR (beta receptor blocker[Title/Abstract])) OR (beta receptor blocking agent[Title/Abstract])) OR (beta sympathicolytic agent[Title/Abstract])) OR (beta sympathicolytics[Title/Abstract])) OR (beta sympatholytic agent[Title/Abstract])) OR (betasympatholytic agent[Title/Abstract])) OR (beta adrenergic receptor blocking agent[Title/Abstract])) OR (carvedilol[Title/Abstract])) OR (bisoprolol[Title/Abstract])) OR (nebivolol[Title/Abstract])) OR (metoprolol[Title/Abstract]) | "adrenergic beta antagonists"[MeSH Terms] OR "adrenergic beta antagonist*"[Title/Abstract] OR "adrenergic beta receptor blockader*"[Title/Abstract] OR "beta adrenergic antagonist*"[Title/Abstract] OR "beta adrenoceptor antagonist*"[Title/Abstract] OR "beta adrenergic blocking agent*"[Title/Abstract] OR "beta adrenergic receptor blockader*"[Title/Abstract] OR "beta adrenergic blocker*"[Title/Abstract] OR "beta adrenoceptor blocking drug"[Title/Abstract] OR "beta adrenolytic"[Title/Abstract] OR "beta adrenolytic agent"[Title/Abstract] OR ((("beta"[Journal] OR "beta"[All Fields]) AND ("block"[All Fields] OR "blocked"[All Fields] OR "Blocking"[All Fields] OR "blockings"[All Fields] OR "blocks"[All Fields])) AND "adrenergic agent"[Title/Abstract]) OR "beta blocking agent"[Title/Abstract] OR "beta blocking drug"[Title/Abstract] OR "beta receptor blocker"[Title/Abstract] OR "beta receptor blocking agent"[Title/Abstract] OR (("beta"[Journal] OR "beta"[All Fields]) AND "sympathicolytic agent"[Title/Abstract]) OR "beta sympathicolytics"[Title/Abstract] OR "beta sympatholytic agent"[Title/Abstract] OR ("betasympatholytic"[All Fields] AND "agent"[Title/Abstract]) OR "beta adrenergic receptor blocking agent"[Title/Abstract] OR "carvedilol"[Title/Abstract] OR "bisoprolol"[Title/Abstract] OR "nebivolol"[Title/Abstract] OR "metoprolol"[Title/Abstract] | 57,697 |
| 2 | (((((Anthracyclines[MeSH Terms]) OR (Anthracycline*[Title/Abstract])) OR (anthracyclin[Title/Abstract])) OR (Daunorubicin[Title/Abstract])) OR (epirubicin[Title/Abstract])) OR (Aclarubicin[Title/Abstract]) | "anthracyclines"[MeSH Terms] OR "anthracycline*"[Title/Abstract] OR "anthracyclin"[Title/Abstract] OR "Daunorubicin"[Title/Abstract] OR "epirubicin"[Title/Abstract] OR "Aclarubicin"[Title/Abstract] | 95,517 |
| 3 | (((Trastuzumab[MeSH Terms]) OR (Trastuzumab[Title/Abstract])) OR (Herceptin[Title/Abstract])) OR (Trazimera[Title/Abstract]) | "Trastuzumab"[MeSH Terms] OR "Trastuzumab"[Title/Abstract] OR "Herceptin"[Title/Abstract] OR "Trazimera"[Title/Abstract] | 17,447 |
| 4 | ((((((Cardiotoxicity[MeSH Terms]) OR (Cardiotoxicit*[Title/Abstract])) OR (Cardiac Toxicit*[Title/Abstract])) OR (cardio toxicity[Title/Abstract])) OR (cardiotoxic effect[Title/Abstract])) OR (cardiotoxicology[Title/Abstract])) OR (heart toxicity[Title/Abstract]) | "cardiotoxicity"[MeSH Terms] OR "cardiotoxicit*"[Title/Abstract] OR "cardiac toxicit*"[Title/Abstract] OR "cardio toxicity"[Title/Abstract] OR "cardiotoxic effect"[Title/Abstract] OR "cardiotoxicology"[Title/Abstract] OR "heart toxicity"[Title/Abstract] | 23,679 |
| 5 | ((Ventricular Dysfunction[MeSH Terms]) OR (Ventricular Dysfunction*[Title/Abstract])) OR (heart ventricle function[Title/Abstract]) | "ventricular dysfunction"[MeSH Terms] OR "ventricular dysfunction*"[Title/Abstract] OR (("heart"[MeSH Terms] OR "heart"[All Fields] OR "hearts"[All Fields] OR "heart s"[All Fields]) AND "ventricle function"[Title/Abstract]) | 59,507 |
| 6 | (((((((((((((((((((((Heart Failure[MeSH Terms]) OR (Heart Failure[Title/Abstract])) OR (Cardiac Failure[Title/Abstract])) OR (Heart Decompensation[Title/Abstract])) OR (Congestive Heart Failure[Title/Abstract])) OR (Right Sided Heart Failure[Title/Abstract])) OR (Left Sided Heart Failure[Title/Abstract])) OR (Myocardial Failure[Title/Abstract])) OR (cardiac backward failure[Title/Abstract])) OR (cardiac decompensation[Title/Abstract])) OR (cardiac incompetence[Title/Abstract])) OR (cardiac insufficiency[Title/Abstract])) OR (cardiac stand still[Title/Abstract])) OR (cardial decompensation[Title/Abstract])) OR (cardial insufficiency[Title/Abstract])) OR (chronic heart insufficiency[Title/Abstract])) OR (decompensatio cordis[Title/Abstract])) OR (heart backward failure[Title/Abstract])) OR (heart incompetence[Title/Abstract])) OR (heart insufficiency[Title/Abstract])) OR (insufficientia cardis[Title/Abstract])) OR (myocardial insufficiency[Title/Abstract]) | "heart failure"[MeSH Terms] OR "heart failure"[Title/Abstract] OR "cardiac failure"[Title/Abstract] OR "heart decompensation"[Title/Abstract] OR "congestive heart failure"[Title/Abstract] OR "right sided heart failure"[Title/Abstract] OR "left sided heart failure"[Title/Abstract] OR "myocardial failure"[Title/Abstract] OR (("cardiacs"[All Fields] OR "Heart"[MeSH Terms] OR "Heart"[All Fields] OR "Cardiac"[All Fields]) AND "backward failure"[Title/Abstract]) OR "cardiac decompensation"[Title/Abstract] OR "cardiac incompetence"[Title/Abstract] OR "cardiac insufficiency"[Title/Abstract] OR (("cardiacs"[All Fields] OR "Heart"[MeSH Terms] OR "Heart"[All Fields] OR "Cardiac"[All Fields]) AND "stand still"[Title/Abstract]) OR "cardial decompensation"[Title/Abstract] OR "cardial insufficiency"[Title/Abstract] OR "chronic heart insufficiency"[Title/Abstract] OR ("decompensatio"[All Fields] AND "cordis"[Title/Abstract]) OR (("Heart"[MeSH Terms] OR "Heart"[All Fields] OR "hearts"[All Fields] OR "heart s"[All Fields]) AND "backward failure"[Title/Abstract]) OR (("Heart"[MeSH Terms] OR "Heart"[All Fields] OR "hearts"[All Fields] OR "heart s"[All Fields]) AND "incompetence"[Title/Abstract]) OR "heart insufficiency"[Title/Abstract] OR ("insufficientia"[All Fields] AND "cardis"[Title/Abstract]) OR "myocardial insufficiency"[Title/Abstract] | 306,574 |
| 7 | ((((((Anthracyclines[MeSH Terms]) OR (Anthracycline*[Title/Abstract])) OR (anthracyclin[Title/Abstract])) OR (Daunorubicin[Title/Abstract])) OR (epirubicin[Title/Abstract])) OR (Aclarubicin[Title/Abstract])) OR ((((Trastuzumab[MeSH Terms]) OR (Trastuzumab[Title/Abstract])) OR (Herceptin[Title/Abstract])) OR (Trazimera[Title/Abstract])) | "anthracyclines"[MeSH Terms] OR "anthracycline*"[Title/Abstract] OR "anthracyclin"[Title/Abstract] OR "Daunorubicin"[Title/Abstract] OR "epirubicin"[Title/Abstract] OR "Aclarubicin"[Title/Abstract] OR "Trastuzumab"[MeSH Terms] OR "Trastuzumab"[Title/Abstract] OR "Herceptin"[Title/Abstract] OR "Trazimera"[Title/Abstract] | 110,837 |
| 8 | ((((((((Cardiotoxicity[MeSH Terms]) OR (Cardiotoxicit*[Title/Abstract])) OR (Cardiac Toxicit*[Title/Abstract])) OR (cardio toxicity[Title/Abstract])) OR (cardiotoxic effect[Title/Abstract])) OR (cardiotoxicology[Title/Abstract])) OR (heart toxicity[Title/Abstract])) OR (((Ventricular Dysfunction[MeSH Terms]) OR (Ventricular Dysfunction*[Title/Abstract])) OR (heart ventricle function[Title/Abstract]))) OR ((((((((((((((((((((((Heart Failure[MeSH Terms]) OR (Heart Failure[Title/Abstract])) OR (Cardiac Failure[Title/Abstract])) OR (Heart Decompensation[Title/Abstract])) OR (Congestive Heart Failure[Title/Abstract])) OR (Right Sided Heart Failure[Title/Abstract])) OR (Left Sided Heart Failure[Title/Abstract])) OR (Myocardial Failure[Title/Abstract])) OR (cardiac backward failure[Title/Abstract])) OR (cardiac decompensation[Title/Abstract])) OR (cardiac incompetence[Title/Abstract])) OR (cardiac insufficiency[Title/Abstract])) OR (cardiac stand still[Title/Abstract])) OR (cardial decompensation[Title/Abstract])) OR (cardial insufficiency[Title/Abstract])) OR (chronic heart insufficiency[Title/Abstract])) OR (decompensatio cordis[Title/Abstract])) OR (heart backward failure[Title/Abstract])) OR (heart incompetence[Title/Abstract])) OR (heart insufficiency[Title/Abstract])) OR (insufficientia cardis[Title/Abstract])) OR (myocardial insufficiency[Title/Abstract])) | "cardiotoxicity"[MeSH Terms] OR "cardiotoxicit*"[Title/Abstract] OR "cardiac toxicit*"[Title/Abstract] OR "cardio toxicity"[Title/Abstract] OR "cardiotoxic effect"[Title/Abstract] OR "cardiotoxicology"[Title/Abstract] OR "heart toxicity"[Title/Abstract] OR ("ventricular dysfunction"[MeSH Terms] OR "ventricular dysfunction*"[Title/Abstract] OR (("Heart"[MeSH Terms] OR "Heart"[All Fields] OR "hearts"[All Fields] OR "heart s"[All Fields]) AND "ventricle function"[Title/Abstract])) OR ("heart failure"[MeSH Terms] OR "heart failure"[Title/Abstract] OR "cardiac failure"[Title/Abstract] OR "heart decompensation"[Title/Abstract] OR "congestive heart failure"[Title/Abstract] OR "right sided heart failure"[Title/Abstract] OR "left sided heart failure"[Title/Abstract] OR "myocardial failure"[Title/Abstract] OR (("cardiacs"[All Fields] OR "Heart"[MeSH Terms] OR "Heart"[All Fields] OR "Cardiac"[All Fields]) AND "backward failure"[Title/Abstract]) OR "cardiac decompensation"[Title/Abstract] OR "cardiac incompetence"[Title/Abstract] OR "cardiac insufficiency"[Title/Abstract] OR (("cardiacs"[All Fields] OR "Heart"[MeSH Terms] OR "Heart"[All Fields] OR "Cardiac"[All Fields]) AND "stand still"[Title/Abstract]) OR "cardial decompensation"[Title/Abstract] OR "cardial insufficiency"[Title/Abstract] OR "chronic heart insufficiency"[Title/Abstract] OR ("decompensatio"[All Fields] AND "cordis"[Title/Abstract]) OR (("Heart"[MeSH Terms] OR "Heart"[All Fields] OR "hearts"[All Fields] OR "heart s"[All Fields]) AND "backward failure"[Title/Abstract]) OR (("Heart"[MeSH Terms] OR "Heart"[All Fields] OR "hearts"[All Fields] OR "heart s"[All Fields]) AND "incompetence"[Title/Abstract]) OR "heart insufficiency"[Title/Abstract] OR ("insufficientia"[All Fields] AND "cardis"[Title/Abstract]) OR "myocardial insufficiency"[Title/Abstract]) | 364,193 |
| 9 | ((((((((((((((((((((((((((Adrenergic beta-Antagonists[MeSH Terms]) OR (Adrenergic beta Antagonist*[Title/Abstract])) OR (Adrenergic beta Receptor Blockader*[Title/Abstract])) OR (beta Adrenergic Antagonist*[Title/Abstract])) OR (beta Adrenoceptor Antagonist*[Title/Abstract])) OR (beta Adrenergic Blocking Agent*[Title/Abstract])) OR (beta Adrenergic Receptor Blockader*[Title/Abstract])) OR (beta Adrenergic Blocker*[Title/Abstract])) OR (beta adrenoceptor blocking drug[Title/Abstract])) OR (beta adrenolytic[Title/Abstract])) OR (beta adrenolytic agent[Title/Abstract])) OR (beta blocking adrenergic agent[Title/Abstract])) OR (beta blocking agent[Title/Abstract])) OR (beta blocking drug[Title/Abstract])) OR (beta receptor blocker[Title/Abstract])) OR (beta receptor blocking agent[Title/Abstract])) OR (beta sympathicolytic agent[Title/Abstract])) OR (beta sympathicolytics[Title/Abstract])) OR (beta sympatholytic agent[Title/Abstract])) OR (betasympatholytic agent[Title/Abstract])) OR (beta adrenergic receptor blocking agent[Title/Abstract])) OR (carvedilol[Title/Abstract])) OR (bisoprolol[Title/Abstract])) OR (nebivolol[Title/Abstract])) OR (metoprolol[Title/Abstract])) AND (((((((Anthracyclines[MeSH Terms]) OR (Anthracycline*[Title/Abstract])) OR (anthracyclin[Title/Abstract])) OR (Daunorubicin[Title/Abstract])) OR (epirubicin[Title/Abstract])) OR (Aclarubicin[Title/Abstract])) OR ((((Trastuzumab[MeSH Terms]) OR (Trastuzumab[Title/Abstract])) OR (Herceptin[Title/Abstract])) OR (Trazimera[Title/Abstract])))) AND (((((((((Cardiotoxicity[MeSH Terms]) OR (Cardiotoxicit*[Title/Abstract])) OR (Cardiac Toxicit*[Title/Abstract])) OR (cardio toxicity[Title/Abstract])) OR (cardiotoxic effect[Title/Abstract])) OR (cardiotoxicology[Title/Abstract])) OR (heart toxicity[Title/Abstract])) OR (((Ventricular Dysfunction[MeSH Terms]) OR (Ventricular Dysfunction*[Title/Abstract])) OR (heart ventricle function[Title/Abstract]))) OR ((((((((((((((((((((((Heart Failure[MeSH Terms]) OR (Heart Failure[Title/Abstract])) OR (Cardiac Failure[Title/Abstract])) OR (Heart Decompensation[Title/Abstract])) OR (Congestive Heart Failure[Title/Abstract])) OR (Right Sided Heart Failure[Title/Abstract])) OR (Left Sided Heart Failure[Title/Abstract])) OR (Myocardial Failure[Title/Abstract])) OR (cardiac backward failure[Title/Abstract])) OR (cardiac decompensation[Title/Abstract])) OR (cardiac incompetence[Title/Abstract])) OR (cardiac insufficiency[Title/Abstract])) OR (cardiac stand still[Title/Abstract])) OR (cardial decompensation[Title/Abstract])) OR (cardial insufficiency[Title/Abstract])) OR (chronic heart insufficiency[Title/Abstract])) OR (decompensatio cordis[Title/Abstract])) OR (heart backward failure[Title/Abstract])) OR (heart incompetence[Title/Abstract])) OR (heart insufficiency[Title/Abstract])) OR (insufficientia cardis[Title/Abstract])) OR (myocardial insufficiency[Title/Abstract]))) | ("adrenergic beta antagonists"[MeSH Terms] OR "adrenergic beta antagonist*"[Title/Abstract] OR "adrenergic beta receptor blockader*"[Title/Abstract] OR "beta adrenergic antagonist*"[Title/Abstract] OR "beta adrenoceptor antagonist*"[Title/Abstract] OR "beta adrenergic blocking agent*"[Title/Abstract] OR "beta adrenergic receptor blockader*"[Title/Abstract] OR "beta adrenergic blocker*"[Title/Abstract] OR "beta adrenoceptor blocking drug"[Title/Abstract] OR "beta adrenolytic"[Title/Abstract] OR "beta adrenolytic agent"[Title/Abstract] OR ((("beta"[Journal] OR "beta"[All Fields]) AND ("block"[All Fields] OR "blocked"[All Fields] OR "Blocking"[All Fields] OR "blockings"[All Fields] OR "blocks"[All Fields])) AND "adrenergic agent"[Title/Abstract]) OR "beta blocking agent"[Title/Abstract] OR "beta blocking drug"[Title/Abstract] OR "beta receptor blocker"[Title/Abstract] OR "beta receptor blocking agent"[Title/Abstract] OR (("beta"[Journal] OR "beta"[All Fields]) AND "sympathicolytic agent"[Title/Abstract]) OR "beta sympathicolytics"[Title/Abstract] OR "beta sympatholytic agent"[Title/Abstract] OR ("betasympatholytic"[All Fields] AND "agent"[Title/Abstract]) OR "beta adrenergic receptor blocking agent"[Title/Abstract] OR "carvedilol"[Title/Abstract] OR "bisoprolol"[Title/Abstract] OR "nebivolol"[Title/Abstract] OR "metoprolol"[Title/Abstract]) AND ("anthracyclines"[MeSH Terms] OR "anthracycline*"[Title/Abstract] OR "anthracyclin"[Title/Abstract] OR "Daunorubicin"[Title/Abstract] OR "epirubicin"[Title/Abstract] OR "Aclarubicin"[Title/Abstract] OR ("Trastuzumab"[MeSH Terms] OR "Trastuzumab"[Title/Abstract] OR "Herceptin"[Title/Abstract] OR "Trazimera"[Title/Abstract])) AND ("cardiotoxicity"[MeSH Terms] OR "cardiotoxicit*"[Title/Abstract] OR "cardiac toxicit*"[Title/Abstract] OR "cardio toxicity"[Title/Abstract] OR "cardiotoxic effect"[Title/Abstract] OR "cardiotoxicology"[Title/Abstract] OR "heart toxicity"[Title/Abstract] OR ("ventricular dysfunction"[MeSH Terms] OR "ventricular dysfunction*"[Title/Abstract] OR (("Heart"[MeSH Terms] OR "Heart"[All Fields] OR "hearts"[All Fields] OR "heart s"[All Fields]) AND "ventricle function"[Title/Abstract])) OR ("heart failure"[MeSH Terms] OR "heart failure"[Title/Abstract] OR "cardiac failure"[Title/Abstract] OR "heart decompensation"[Title/Abstract] OR "congestive heart failure"[Title/Abstract] OR "right sided heart failure"[Title/Abstract] OR "left sided heart failure"[Title/Abstract] OR "myocardial failure"[Title/Abstract] OR (("cardiacs"[All Fields] OR "Heart"[MeSH Terms] OR "Heart"[All Fields] OR "Cardiac"[All Fields]) AND "backward failure"[Title/Abstract]) OR "cardiac decompensation"[Title/Abstract] OR "cardiac incompetence"[Title/Abstract] OR "cardiac insufficiency"[Title/Abstract] OR (("cardiacs"[All Fields] OR "Heart"[MeSH Terms] OR "Heart"[All Fields] OR "Cardiac"[All Fields]) AND "stand still"[Title/Abstract]) OR "cardial decompensation"[Title/Abstract] OR "cardial insufficiency"[Title/Abstract] OR "chronic heart insufficiency"[Title/Abstract] OR ("decompensatio"[All Fields] AND "cordis"[Title/Abstract]) OR (("Heart"[MeSH Terms] OR "Heart"[All Fields] OR "hearts"[All Fields] OR "heart s"[All Fields]) AND "backward failure"[Title/Abstract]) OR (("Heart"[MeSH Terms] OR "Heart"[All Fields] OR "hearts"[All Fields] OR "heart s"[All Fields]) AND "incompetence"[Title/Abstract]) OR "heart insufficiency"[Title/Abstract] OR ("insufficientia"[All Fields] AND "cardis"[Title/Abstract]) OR "myocardial insufficiency"[Title/Abstract])) | 193 |

**Embase**

| No. | Query | Results |
| --- | --- | --- |
| #1 | 'beta adrenergic receptor blocking agent'/exp OR 'beta adrenergic receptor blocking agent':ti,ab OR 'beta adrenergic antagonist':ti,ab OR 'adrenergic beta antagonist':ti,ab OR 'beta adrenoceptor antagonist':ti,ab OR 'beta adrenergic blocking agent':ti,ab OR 'beta adrenergic receptor blockader':ti,ab OR 'beta adrenergic blocker':ti,ab OR 'beta adrenoceptor blocking drug':ti,ab OR 'beta adrenolytic':ti,ab OR 'beta adrenolytic agent':ti,ab OR 'beta blocking adrenergic agent':ti,ab OR 'beta blocking agent':ti,ab OR 'beta blocking drug':ti,ab OR 'beta receptor blocker':ti,ab OR 'beta receptor blocking agent':ti,ab OR 'beta sympathicolytic agent':ti,ab OR 'beta sympathicolytics':ti,ab OR 'beta sympatholytic agent':ti,ab OR 'betasympatholytic agent':ti,ab OR 'carvedilol'/exp OR 'carvedilol':ti,ab OR 'bisoprolol'/exp OR 'bisoprolol':ti,ab OR 'nebivolol'/exp OR 'nebivolol':ti,ab OR 'metoprolol'/exp OR 'metoprolol':ti,ab | 382261 |
| #2 | 'anthracycline'/exp OR 'anthracycline*':ti,ab OR 'anthracyclin':ti,ab OR 'daunorubicin'/exp OR 'daunorubicin':ti,ab OR 'epirubicin'/exp OR 'epirubicin':ti,ab OR 'aclarubicin'/exp OR 'aclarubicin':ti,ab | 106196 |
| #3 | 'trastuzumab'/exp OR 'trastuzumab':ti,ab OR 'herceptin':ti,ab OR 'trazimera':ti,ab | 61499 |
| #4 | 'cardiotoxicity'/exp OR 'cardiotoxicit*':ti,ab OR 'cardiac toxicit*':ti,ab OR 'cardio toxicity':ti,ab OR 'cardiotoxic effect':ti,ab OR 'cardiotoxicology':ti,ab OR 'heart toxicity':ti,ab | 66601 |
| #5 | 'ventricular dysfunction'/exp OR 'ventricular dysfunction*':ti,ab OR 'heart ventricle function':ti,ab | 109878 |
| #6 | 'heart failure'/exp OR 'heart failure':ti,ab OR 'cardiac failure':ti,ab OR 'heart decompensation':ti,ab OR 'congestive heart failure':ti,ab OR 'right sided heart failure':ti,ab OR 'left sided heart failure':ti,ab OR 'myocardial failure':ti,ab OR 'cardiac backward failure':ti,ab OR 'cardiac decompensation':ti,ab OR 'cardiac incompetence':ti,ab OR 'cardiac insufficiency':ti,ab OR 'cardiac stand still':ti,ab OR 'cardial decompensation':ti,ab OR 'cardial insufficiency':ti,ab OR 'chronic heart insufficiency':ti,ab OR 'decompensatio cordis':ti,ab OR 'heart backward failure':ti,ab OR 'heart incompetence':ti,ab OR 'heart insufficiency':ti,ab OR 'insufficientia cardis':ti,ab OR 'myocardial insufficiency':ti,ab | 854165 |
| #7 | #2 OR #3 | 155811 |
| #8 | #4 OR #5 OR #6 | 960296 |

**Cochrane**

| ID | Search | Hits |
| --- | --- | --- |
| #1 | MeSH descriptor: [Adrenergic beta-Antagonists] explode all trees | 5677 |
| #2 | ((Adrenergic beta Antagonist*):ti,ab,kw) OR ((Adrenergic beta Receptor Blockader*):ti,ab,kw) OR ((beta Adrenergic Antagonist*):ti,ab,kw) OR ((beta Adrenoceptor Antagonist*):ti,ab,kw) OR ((beta Adrenergic Blocking Agent*):ti,ab,kw) OR ((beta Adrenergic Receptor Blockader*):ti,ab,kw) OR ((beta Adrenergic Blocker*):ti,ab,kw) OR ((beta adrenoceptor blocking drug):ti,ab,kw) OR ((beta adrenolytic):ti,ab,kw) OR ((beta adrenolytic agent):ti,ab,kw) OR ((beta blocking adrenergic agent):ti,ab,kw) OR ((beta blocking agent):ti,ab,kw) OR ((beta blocking drug):ti,ab,kw) OR ((beta receptor blocker):ti,ab,kw) OR ((beta receptor blocking agent):ti,ab,kw) OR ((beta sympathicolytic agent):ti,ab,kw) OR ((beta sympathicolytics):ti,ab,kw) OR ((beta sympatholytic agent):ti,ab,kw) OR ((betasympatholytic agent):ti,ab,kw) OR ((beta adrenergic receptor blocking agent):ti,ab,kw) OR ((carvedilol):ti,ab,kw) OR ((bisoprolol):ti,ab,kw) OR ((nebivolol):ti,ab,kw) | 13790 |
| #3 | #1 OR #2 | 13790 |
| #4 | MeSH descriptor: [Anthracyclines] explode all trees | 7077 |
| #5 | ((Anthracycline*):ti,ab,kw) OR ((anthracyclin):ti,ab,kw) OR ((Daunorubicin):ti,ab,kw) OR ((epirubicin):ti,ab,kw) OR ((Aclarubicin):ti,ab,kw) | 8135 |
| #6 | #4 OR #5 | 12357 |
| #7 | MeSH descriptor: [Trastuzumab] explode all trees | 1306 |
| #8 | (Trastuzumab):ti,ab,kw OR (Herceptin):ti,ab,kw OR (Trazimera):ti,ab,kw | 4031 |
| #9 | #7 OR #8 | 4031 |
| #10 | MeSH descriptor: [Cardiotoxicity] explode all trees | 237 |
| #11 | (Cardiotoxicit*):ti,ab,kw OR (Cardiac Toxicit*):ti,ab,kw OR (cardio toxicity):ti,ab,kw OR (cardiotoxic effect):ti,ab,kw OR (cardiotoxicology):ti,ab,kw | 4178 |
| #12 | (heart toxicity):ti,ab,kw | 3239 |
| #13 | #10 OR #11 OR #12 | 6121 |
| #14 | MeSH descriptor: [Ventricular Dysfunction] explode all trees | 2915 |
| #15 | (Ventricular Dysfunction*):ti,ab,kw OR (heart ventricle function):ti,ab,kw | 12071 |
| #16 | #14 OR #15 | 12090 |
| #17 | MeSH descriptor: [Heart Failure] explode all trees | 15009 |
| #18 | ((Heart Failure):ti,ab,kw)OR ((Cardiac Failure):ti,ab,kw)OR ((Heart Decompensation):ti,ab,kw)OR ((Congestive Heart Failure):ti,ab,kw)OR ((Right Sided Heart Failure):ti,ab,kw)OR ((Left Sided Heart Failure):ti,ab,kw)OR ((Myocardial Failure):ti,ab,kw)OR ((cardiac backward failure):ti,ab,kw)OR ((cardiac decompensation):ti,ab,kw)OR ((cardiac incompetence):ti,ab,kw)OR ((cardiac insufficiency):ti,ab,kw)OR ((cardiac stand still):ti,ab,kw)OR ((cardial decompensation):ti,ab,kw)OR ((cardial insufficiency):ti,ab,kw)OR ((chronic heart insufficiency):ti,ab,kw)OR ((decompensatio cordis):ti,ab,kw)OR ((heart backward failure):ti,ab,kw)OR ((heart incompetence):ti,ab,kw)OR ((heart insufficiency):ti,ab,kw)OR ((insufficientia cardis):ti,ab,kw)OR ((myocardial insufficiency):ti,ab,kw) | 54815 |
| #19 | #17 OR #18 | 54843 |
| #20 | #6 OR #9 | 15547 |
| #21 | #13 OR #16 OR #19 | 64618 |
| #22 | #3 AND #20 AND #21 | 125 |

**Wos**

| # | Search Query | Results |
| --- | --- | --- |
| 9 | TS=(Adrenergic beta-Antagonists) OR TS=(Adrenergic beta Antagonist*) OR TS=(Adrenergic beta Receptor Blockader*) OR TS=(beta Adrenergic Antagonist*) OR TS=(beta Adrenoceptor Antagonist*) OR TS=(beta Adrenergic Blocking Agent*) OR TS=(beta Adrenergic Receptor Blockader*) OR TS=(beta Adrenergic Blocker*) OR TS=(beta adrenoceptor blocking drug) OR TS=(beta adrenolytic) OR TS=(beta adrenolytic agent) OR TS=(beta blocking adrenergic agent) OR TS=(beta blocking agent) OR TS=(beta blocking drug) OR TS=(beta receptor blocker) OR TS=(beta receptor blocking agent) OR TS=(beta sympathicolytic agent) OR TS=(beta sympathicolytics) OR TS=(beta sympatholytic agent) OR TS=(betasympatholytic agent) OR TS=(beta adrenergic receptor blocking agent) OR TS=(carvedilol) OR TS=(bisoprolol) OR TS=(nebivolol) OR TS=(metoprolol) | 58666 |
| 10 | ((((TS=(Anthracycline*)) OR TS=(anthracyclin)) OR TS=(Daunorubicin)) OR TS=(epirubicin)) OR TS=(Aclarubicin) | 34086 |
| 11 | ((TS=(Trastuzumab )) OR TS=(Herceptin)) OR TS=(Trazimera) | 28840 |
| 12 | (((((TS=(Cardiotoxicit*)) OR TS=(Cardiac Toxicit*)) OR TS=(cardio toxicity)) OR TS=(cardiotoxic effect)) OR TS=(cardiotoxicology)) OR TS=(heart toxicity) | 51626 |
| 13 | (TS=(Ventricular Dysfunction*)) OR TS=(heart ventricle function) | 104707 |
| 14 | TS=(Heart Failure) OR TS=("Cardiac Failure") OR TS=("Heart Decompensation") OR TS=("Congestive Heart Failure") OR TS=("Right Sided Heart Failure") OR TS=("Left Sided Heart Failure") OR TS=("Myocardial Failure") OR TS=("cardiac backward failure") OR TS=("cardiac decompensation") OR TS=("cardiac incompetence") OR TS=("cardiac insufficiency") OR TS=("cardiac stand still") OR TS=("cardial decompensation") OR TS=("cardial insufficiency") OR TS=("chronic heart insufficiency") OR TS=("decompensatio cordis") OR TS=("heart backward failure") OR TS=("heart incompetence") OR TS=("heart insufficiency") OR TS=("insufficientia cardis") OR TS=("myocardial insufficiency") | 397763 |
| 18 | #10 OR #11 | 60522 |
| 19 | #12 OR #13 OR #14 | 493247 |
| 20 | #9 AND #18 AND #19 | 265 |
