# Supplementary material for: Protective effects of beta-blockers against anthracycline- and trastuzumab-related cardiotoxicity: a systematic review based on conventional and Bayesian network meta-analysis
Source: Front Cardiovasc Med. 2026 Apr 1;13:1777908. doi: 10.3389/fcvm.2026.1777908 (PMC13079159; doi:10.3389/fcvm.2026.1777908)
Supplement: Supplementary Material S2 — Details of Network Meta-Analysis. [file Table2.docx]

**Convergence Assessment:**

To ensure the reliability of the network meta-analysis results, we assessed the convergence of the Markov Chain Monte Carlo (MCMC) simulations using Gelman-Rubin statistics (R-hat). As specific values were not available, Gelman-Rubin diagnostic plots were generated to visually inspect the convergence of MCMC chains. Convergence was considered satisfactory when the diagnostic plots showed that the chains had stabilized and the R-hat values approached 1.0 for all parameters.

Gelman-Rubin Diagnostic Plot for Convergence

This plot shows the diagnostic of MCMC chain convergence. The stabilization of the chains indicates that the MCMC simulation has converged satisfactorily.


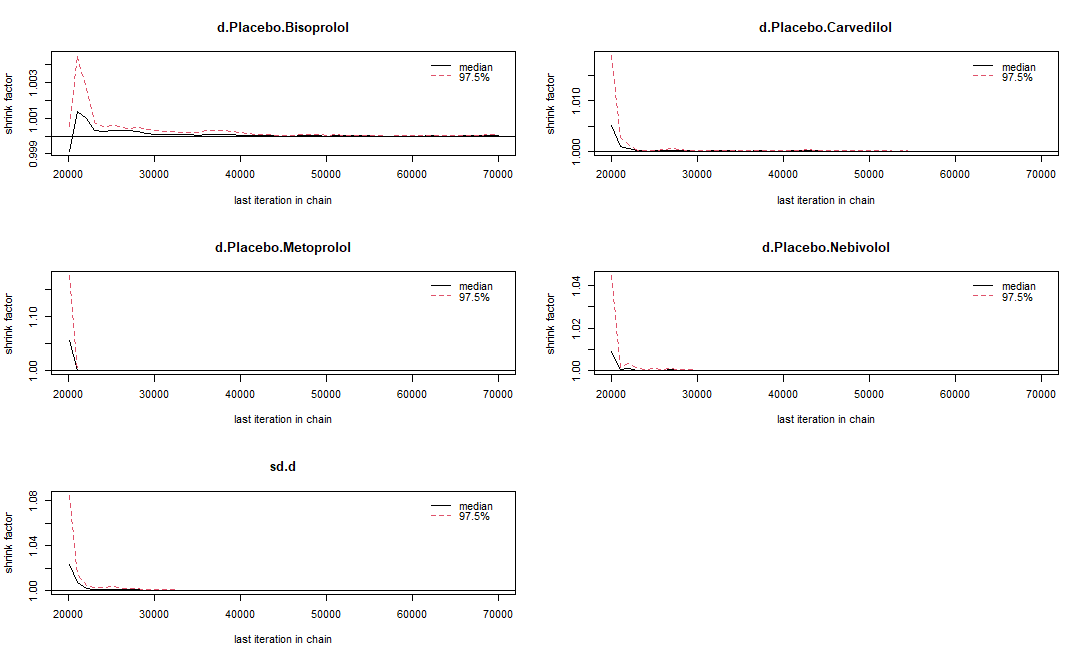


Convergence Diagnosis for LVEF


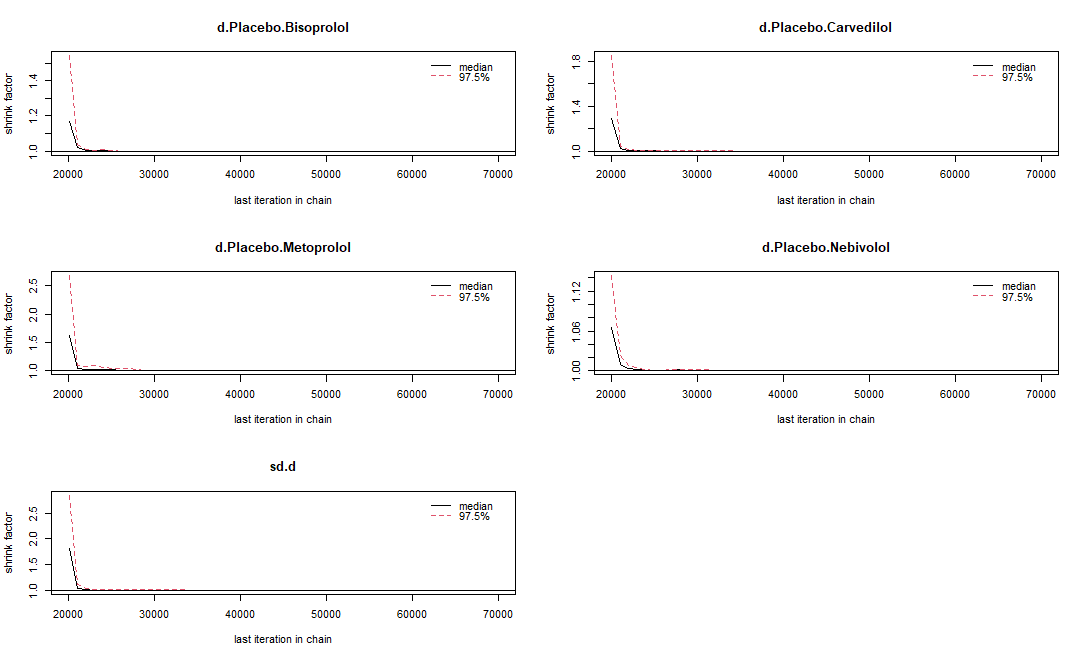


Convergence Diagnosis for CTRCE

**Prior Distribution Settings:**

We used non-informative prior distributions for all parameters, including:

For treatment effects: Normal distribution with mean = 0 and variance = 100. For between-study variance: Half-normal distribution with mean = 0 and variance = 1.

**Chain Length:**

We ran 4 MCMC chains, each with 50,000 iterations and a burn-in period of 20,000 iterations. The convergence of the chains was visually assessed using Gelman-Rubin statistics, and convergence was confirmed when the diagnostic plots showed stable results.
