# Supplementary figures and images for: Protective effects of beta-blockers against anthracycline- and trastuzumab-related cardiotoxicity: a systematic review based on conventional and Bayesian network meta-analysis
Source: Front Cardiovasc Med. 2026 Apr 1;13:1777908. doi: 10.3389/fcvm.2026.1777908 (PMC13079159; doi:10.3389/fcvm.2026.1777908)

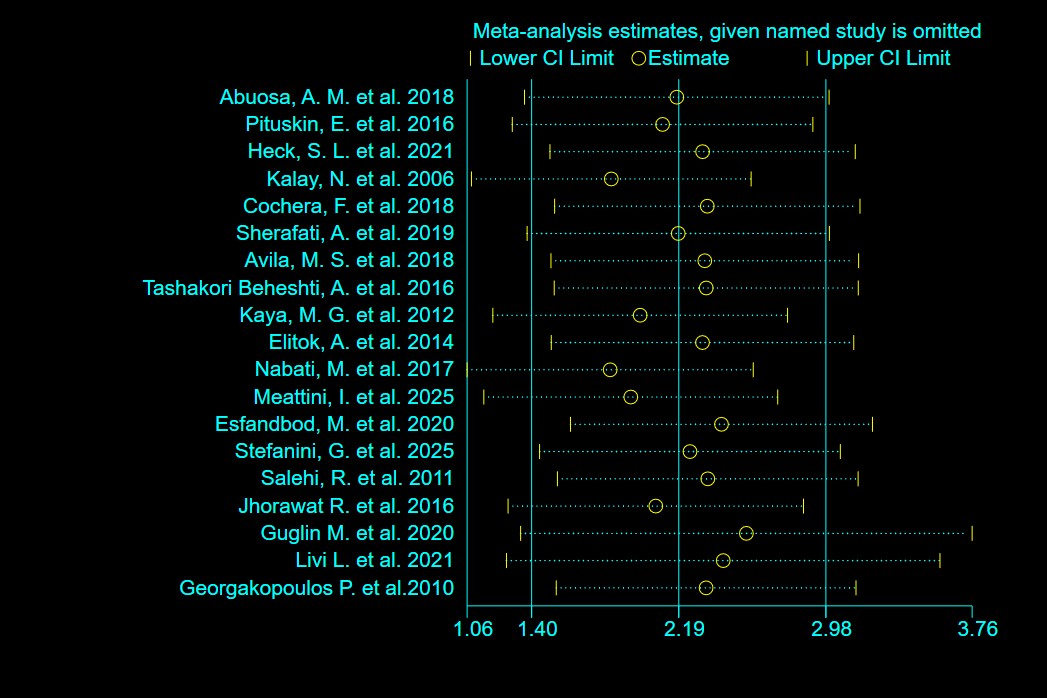

Supplement: Supplementary Material S7 — Funnel plot for LVEF network meta-analysis. [file Image1.jpeg]

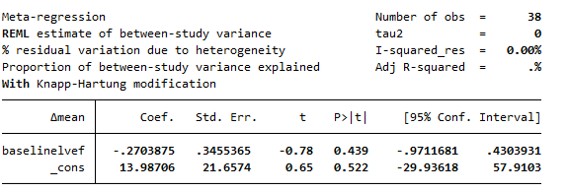

Supplement: Supplementary Material S8 — Sensitivity analysis for CTRCE incidence. [file Image2.jpeg]

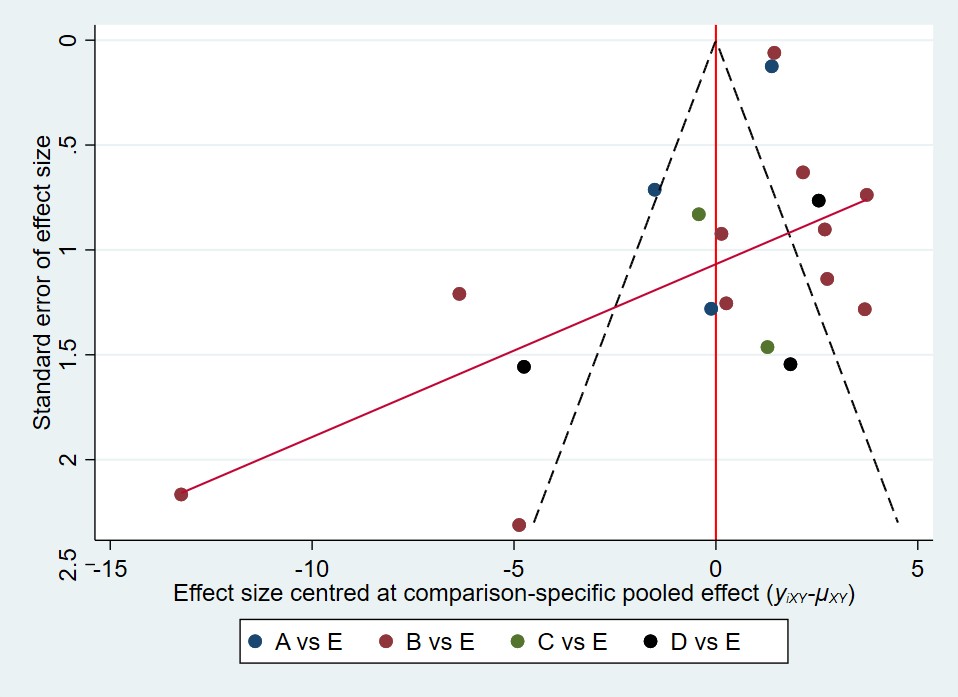

Supplement: Supplementary Material S9 — Egger's test for publication bias in traditional meta-analysis of CTRCE. [file Image3.jpeg]

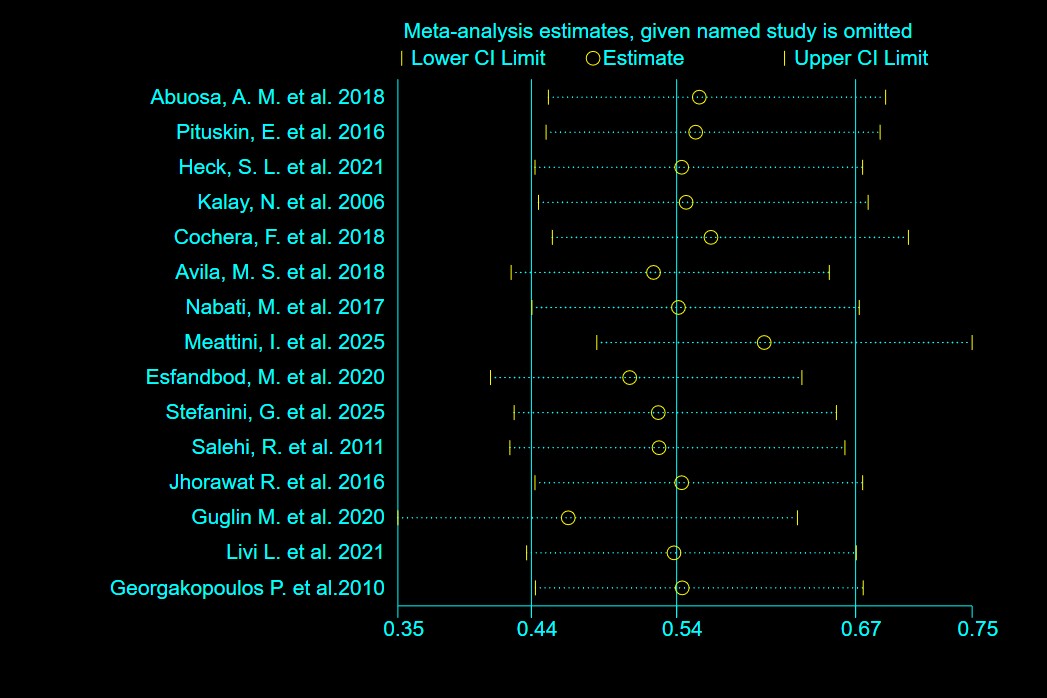

Supplement: Supplementary Material S10 — DIC results for CTRCE network meta-analysis. [file Image4.jpeg]

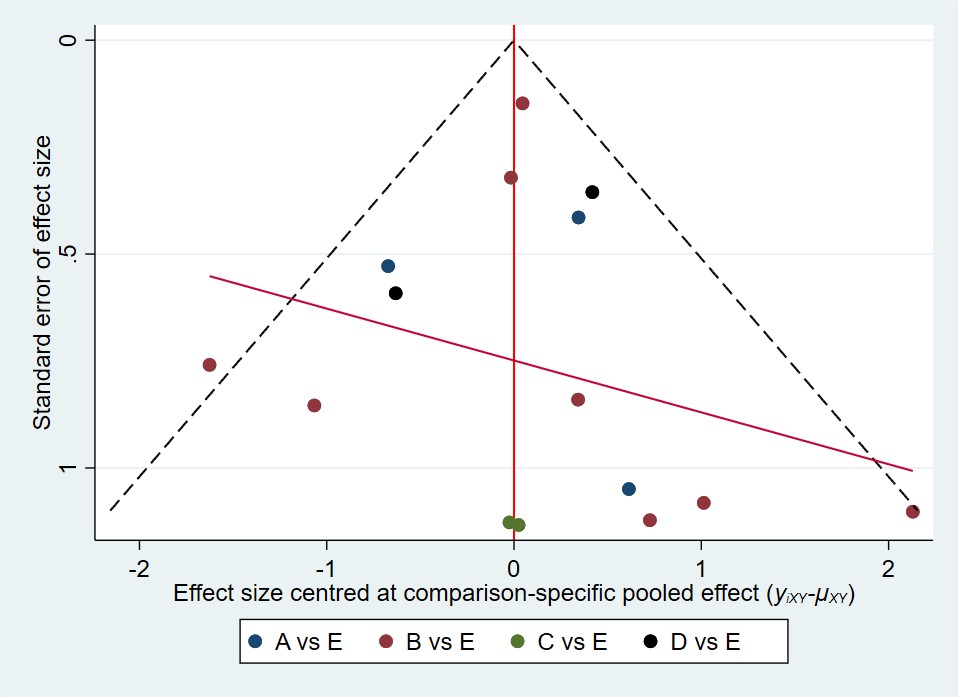

Supplement: Supplementary Material S11 — Funnel plot for CTRCE network meta-analysis. [file Image5.jpeg]
